# Selective Cytotoxic Activity of Derivatives of the Natural Alkaloid Tetrandrine Against Cancer Cell Lines: In Vitro and In Silico Studies

**DOI:** 10.3390/molecules31193572

**Published:** 2026-10-08

**Authors:** María Daniela Alvarez-Campa, Carmen María López-Saiz, Héctor Enrique Trujillo-Ruiz, Carmen Lizette Del-Toro-Sánchez, Juan Carlos Gálvez-Ruiz, Ramón Alfonso Moreno-Corral, Maribel Plascencia-Jatomea, Ariel Humberto Soto-Sánchez, Erika Silva-Campa, Martin S. Hernández-Zazueta, Karen Ochoa Lara

**Affiliations:** 1 Departamento de Investigación y Posgrado en Alimentos, Universidad de Sonora, Rosales y Encinas s/n, Colonia Centro, Hermosillo 83000, Sonora, Mexico; a220213558@unison.mx (M.D.A.-C.); carmen.lopezsaiz@unison.mx (C.M.L.-S.); a213204436@unison.mx (H.E.T.-R.); carmen.deltoro@unison.mx (C.L.D.-T.-S.); maribel.plascencia@unison.mx (M.P.-J.); 2 Departamento de Ciencias Químico-Biológicas, Universidad de Sonora, Rosales y Encinas s/n, Colonia Centro, Hermosillo 83000, Sonora, Mexico; juan.galvez@unison.mx; 3 Departamento de Investigación en Polímeros y Materiales, Universidad de Sonora, Rosales y Encinas s/n, Colonia Centro, Hermosillo 83000, Sonora, Mexico; ramonalfonso.moreno@unison.mx (R.A.M.-C.); a220217798@unison.mx (A.H.S.-S.); 4 Departamento de Física, Universidad de Sonora, Rosales y Encinas s/n, Colonia Centro, Hermosillo 83000, Sonora, Mexico; erika.silva@unison.mx

**Keywords:** tetrandrine, tetrandrine derivatives, selective cytotoxicity, cancer cell lines, molecular docking, anticancer agents

## Abstract

**Tetrandrine** (**TET**) is a bisbenzylisoquinoline alkaloid with promising anticancer activity, although its limited selectivity toward malignant over non-cancerous cells restricts its therapeutic potential. A series of mono- and bis-N-substituted tetrandrine derivatives was therefore investigated to determine whether N-functionalization could improve the cytotoxic selectivity of the parent scaffold. The series included the two new compounds **MU1NT** and **DPBT**. Following initial screening in non-cancerous ARPE-19 retinal epithelial cells, selected derivatives were evaluated against 22Rv1 prostate carcinoma, A549 lung adenocarcinoma, MDA-MB-231 triple-negative breast cancer, HCT-116 colorectal carcinoma, and HeLa cervical cancer cells. **TET** exhibited broad but poorly selective cytotoxicity, with IC_50_ values of 6.81 ± 1.79 and 6.00 ± 1.96 μM in ARPE-19 and 22Rv1 cells, respectively, corresponding to a selectivity index (*SI*) of 1.13. By contrast, **DBT** showed IC_50_ values of 45.44 ± 1.37 μM in ARPE-19 cells and 9.89 ± 1.08 μM in 22Rv1 cells, with an *SI* of 4.59, the highest experimentally determined value among the tetrandrine-based compounds evaluated. **MU1NT** also displayed improved selectivity toward 22Rv1 cells (*SI* = 2.57), although with lower cytotoxic potency than **DBT**. Fluorescence microscopy using DAPI and phalloidin revealed pronounced nuclear and cytoskeletal alterations in **DBT**-treated 22Rv1 cells, whereas ARPE-19 morphology was comparatively preserved. Annexin V-FITC/PI flow cytometry further demonstrated an apoptosis-associated response in 22Rv1 cells, with total apoptosis reaching 86.30 ± 1.27% for **TET** and 34.77 ± 5.13% for **DBT** after 24 h at their respective MTT-derived IC_50_ concentrations. Molecular docking suggested distinct predicted interaction profiles for **TET** and **DBT** across the apoptosis-related proteins evaluated. Targeted redocking of co-crystallized venetoclax validated the Bcl-2 site-specific protocol (RMSD = 0.55 Å); however, both alkaloids scored less favorably than venetoclax, and the small score difference between **TET** and **DBT** did not account for their distinct cellular selectivity profiles. Collectively, these findings identify **DBT** as a promising tetrandrine derivative with improved selectivity, warranting further mechanistic studies and in vivo evaluation in prostate cancer models.

## 1. Introduction

Cancer remains one of the leading causes of morbidity and mortality worldwide, with an estimated 20 million new cases and 9.7 million cancer-related deaths reported in 2022 [1]. Despite major advances in surgery, radiotherapy, chemotherapy, targeted therapy, and immunotherapy, the efficacy of cancer treatment continues to be limited by systemic toxicity, insufficient tumor selectivity, and the development of intrinsic or acquired drug resistance, which may contribute to treatment failure and disease recurrence [2,3]. Consequently, the identification of anticancer agents capable of preferentially affecting malignant cells while reducing damage to non-cancerous tissues remains an important objective in cancer drug discovery. Natural products and their structurally modified derivatives continue to represent an invaluable source of bioactive molecular scaffolds for anticancer drug discovery owing to their remarkable structural diversity and ability to modulate multiple molecular targets involved in cancer initiation, progression, and therapeutic response [4,5,6].

Tetrandrine is a naturally occurring bisbenzylisoquinoline alkaloid isolated principally from the roots of *Stephania tetrandra* S. Moore. Its macrocyclic structure, formed by two benzylisoquinoline units connected through diaryl ether linkages, provides a conformationally complex scaffold with broad pharmacological potential [3,7,8]. Tetrandrine has shown anticancer activity in a wide variety of experimental models through multiple and sometimes interconnected mechanisms, including inhibition of cell proliferation, induction of cell-cycle arrest, activation of intrinsic and extrinsic apoptotic pathways, modulation of autophagy, generation of reactive oxygen species, suppression of migration and invasion, inhibition of angiogenesis, and reversal of multidrug resistance mediated by P-glycoprotein [3,9,10,11,12,13,14]. These effects have been documented in several human cancer cell lines, including PC-3 and DU145 prostate cancer cells, NPC-TW 076 nasopharyngeal carcinoma cells, and HSC-3 oral squamous cell carcinoma cells [11,12,13,14]. More recently, tetrandrine was shown to activate the STING/TBK1/IRF3 pathway and enhance αPD-1 mAb efficacy in non-small-cell lung cancer by activating the CD8^+^ T cells, further illustrating the diversity of its anticancer mechanisms [15]. In addition, its combination with adriamycin was reported to reverse multidrug resistance in breast cancer cells through modulation of the NLRP3/caspase-1/GSDMD pyroptotic pathway [16]. However, poor aqueous solubility, low oral bioavailability, unfavorable pharmacokinetic properties, and dose-dependent toxicity continue to restrict the broader therapeutic development of the parent alkaloid [3,8,17].

Structural modification of tetrandrine has therefore emerged as an important medicinal-chemistry strategy to improve its potency, selectivity, solubility, metabolic behavior, and overall therapeutic profile [8,17]. Recent efforts have focused predominantly on substitution at the C-5 and C-14 positions, polyhalogenation, modification of the C-12-methoxybenzyl motif, introduction of quaternary ammonium groups, and structural simplification of the bisbenzylisoquinoline framework [8]. C-14 amino acid-, urea-, thiourea-, sulfonamide-, and sulfonate-substituted derivatives have displayed greater antiproliferative activity than tetrandrine against different cancer cell lines [18,19,20]. In particular, sulfonyl-substituted derivatives have shown activity against colon cancer cells, induction of mitochondrial apoptosis, inhibition of the PI3K/AKT/mTOR pathway, and improved selectivity relative to the parent alkaloid [21]. Rational simplification of the tetrandrine scaffold has also yielded potent and selective P-glycoprotein inhibitors capable of reversing multidrug resistance with comparatively low intrinsic cytotoxicity [22]. Furthermore, recent studies on C-7-modified derivatives have expanded the number of positions available for optimization and emphasized the importance of developing clearer structure–activity relationships for tetrandrine analogues [23]. Collectively, these studies demonstrate that structural derivatization can substantially alter the anticancer and pharmacological properties of tetrandrine.

Despite these advances, functionalization of the tertiary nitrogen atoms of tetrandrine has remained comparatively underexplored. Within this area, our research group has systematically developed N-functionalization of tetrandrine over the past 25 years as a versatile strategy for generating structurally diverse mono- and bis-N-substituted quaternary ammonium derivatives bearing aromatic and aliphatic substituents [24,25,26,27,28,29]. Beyond expanding the chemical diversity of the tetrandrine scaffold, these studies have shown that N-functionalization can modulate its physicochemical and biological properties, including aqueous solubility and molecular recognition of biological targets, particularly DNA. Initial biological studies demonstrated that monosubstituted derivatives incorporating 9-methylacridine and 1-methylanthracene moieties at the more reactive nitrogen atom exhibited enhanced DNA-binding affinity and antiproliferative activity [29]. Subsequently, bis-N-substituted derivatives displayed strong interactions with different DNA models while maintaining low cytotoxicity toward non-cancerous cells, supporting the potential of this strategy to generate biologically active compounds with favorable selectivity profiles [30,31,32,33]. Further extension of this approach revealed pronounced antibacterial activity, particularly against *Staphylococcus aureus*, and demonstrated that both the degree of quaternization and the nature of the N-substituents strongly influence biological activity, cytotoxicity, and selectivity [31,32,33]. Taken together, these studies support N-functionalization as a robust platform for tuning the biological properties of tetrandrine and provide a strong rationale for investigating its potential in the development of selective anticancer derivatives. Experimental evidence provides a biological rationale for exploring apoptosis-related proteins as potential molecular recognition partners of tetrandrine. **TET** has been reported to increase DR4 and DR5 expression and sensitize prostate cancer cells to TRAIL-induced apoptosis, while also modulating intrinsic apoptotic signaling regulated by Bcl-2-family proteins [14,34]. Previous computational studies of **TET** have primarily examined kinase-, inflammatory-, and angiogenesis-related targets, including AKT1, the PI3K/AKT axis, EGFR, MMP-13, and SRPK1 [35,36,37]. However, pathway modulation or protein expression does not demonstrate direct ligand binding. Therefore, the apoptosis-related proteins examined here were treated as hypothesis-generating molecular targets, and a complementary site-specific docking protocol for Bcl-2 was validated using its co-crystallized reference inhibitor.

We hypothesized that N-functionalization of the tetrandrine scaffold could modify its cytotoxic profile and enable the identification of derivatives with improved selectivity toward cancer cells. Accordingly, the present study evaluates the cytotoxic activity and selectivity of a series of mono- and bis-N-substituted tetrandrine derivatives (Figure 1) against five human cancer cell lines, 22Rv1 prostate cancer, A549 lung adenocarcinoma, MDA-MB-231 triple-negative breast cancer, HeLa cervical cancer, and HCT-116 colorectal cancer, using non-cancerous ARPE-19 retinal epithelial cells as a comparative model. Cell-viability assays were complemented by fluorescence microscopy and Annexin V-FITC/PI flow cytometry to evaluate treatment-associated morphological alterations and apoptosis-related cell death profiles, respectively. Molecular docking against apoptosis-related proteins, including targeted Bcl-2 docking validated by venetoclax redocking, was used as a hypothesis-generating approach, while public RNA expression data were examined to establish the transcriptional presence of the selected pathway components in the cellular models. This integrated in vitro and in silico approach was designed to identify N-substituted tetrandrine derivatives with enhanced cancer cell selectivity and potential for further development as anticancer candidates.

## 2. Results and Discussion

### 2.1. Synthesis and Structural Characterization

The series of N,N′-disubstituted tetrandrine (**TET**) derivatives **DBT**, **DeBT**, **DIT**, **DNT**, **DQT**, **DAcT** and **DAT**, as well as the monosubstituted derivatives **MAnT** and **MAcT**, were prepared in moderate to good yields, as previously reported in the literature [24,27,29,30,31,33]. The chloroethylurea derivative **U1N** [1-(2-chloroethyl)-3-(naphthalen-1-yl)urea] was synthesized from naphthalen-1-amine and 2-chloroethyl isocyanate following a procedure similar to that described by Johnston et al. (1966) [38].

Similarly, the novel tetrandrine salts **MU1NT** and **DPBT** were synthesized in moderate to good yields through quaternization of one or both tertiary amine groups of the alkaloid with the chloroethylurea derivative **U1N** and 1-bromo-3-phenylpropane, respectively. The 40% yield obtained for **MU1NT** and the 65% yield obtained for **DPBT** fall within the range previously reported for structurally related N-functionalized tetrandrine derivatives, including **DeBT** (40%), **DAcT** (73%), and **DQT** (74%) [33]. Because **MU1NT** and **DPBT** are new compounds, no identical literature values are available for direct comparison. Quaternization of the nitrogen atoms of (S,S)-(+)-tetrandrine generates new stereogenic centers in the resulting derivatives [29,30,31,33]. In all cases, a single set of signals was observed, consistent with the formation of a single stereoisomer and with previous reports. The products were characterized by melting point, IR and NMR (^1^H and ^13^C) spectroscopy, as well as ESI(+) mass spectrometry. The resulting spectroscopic data were consistent with the proposed structures (see Section 3).

The ^1^H and ^13^C NMR spectra of **MU1NT** and **DPBT** each exhibited a single set of signals, further supporting their formation as single stereoisomers. The ^1^H and ^13^C NMR spectra and ESI(+) mass spectra of the new tetrandrine derivatives **MU1NT** and **DPBT** and urea **U1N**, together with the FTIR-ATR spectra of **MU1NT** and **U1N**, are provided in the Supporting Information (Figures S1–S11). Collectively, these spectroscopic data are consistent with the proposed structures and provide strong evidence for the successful synthesis and structural characterization of the novel mono- and disubstituted **TET** derivatives.

### 2.2. In Vitro Cytotoxicity and Selectivity Evaluation

#### 2.2.1. Initial Cytotoxicity Screening of Tetrandrine Derivatives in Non-Cancerous ARPE-19 Cells

As an initial screening step, the cytotoxic effects of the N-substituted tetrandrine derivatives were evaluated in non-cancerous human retinal pigment epithelial ARPE-19 cells. Cells were exposed to each derivative at 25 μM for 48 h, and cell viability was determined using the MTT assay. This experiment was designed as a conservative exclusion filter to identify derivatives with comparatively low cytotoxicity toward non-cancerous cells before evaluating their activity against the panel of human cancer cell lines. Doxorubicin was included as a positive cytotoxic control.

ARPE-19 was selected as a well-characterized non-tumor human epithelial model previously used in our evaluations of tetrandrine derivatives, thereby providing continuity with earlier cytotoxicity studies [32,33]. However, because ARPE-19 is not tissue-matched to the cancer cell lines evaluated, it was used only as a comparative non-cancerous reference model and as a conservative exclusion filter. Accordingly, the calculated selectivity indices describe discrimination between the evaluated cellular models and should not be interpreted as evidence of tissue-specific selectivity.

As shown in Figure 2, the response of ARPE-19 cells varied markedly among the evaluated derivatives. **DBT**, **DeBT**, **DPBT**, **DIT**, **DAT**, and **MU1NT** maintained the highest cell viability percentages and showed significantly greater viability than doxorubicin. Among these compounds, **DBT**, **DeBT**, and **MU1NT** belonged to the highest statistical group, whereas **DPBT**, **DIT**, and **DAT** retained comparatively high viability without significant differences from at least some compounds in that group. In contrast, **DNT** produced a substantially greater reduction in cell viability, while **MAnT** and **MAcT** exhibited the strongest cytotoxic effects under the same experimental conditions. Representative bright-field microscopy images documenting the morphology and confluence of ARPE-19 cells after treatment are provided in Figure S12.

Under the common screening conditions used in ARPE-19 cells (25 μM for 48 h), N-functionalization produced compound-dependent effects on cytotoxicity relative to the parent **TET** scaffold and among the evaluated derivatives. In particular, the pronounced activity of the monosubstituted aromatic derivatives **MAnT** and **MAcT**, previously shown to exhibit antiproliferative activity and strong DNA recognition, contrasts with the comparatively low cytotoxicity of **MU1NT** in ARPE-19 cells [29]. Likewise, the different responses observed among the bis-N-substituted derivatives indicate that their cytotoxic behavior cannot be attributed solely to the degree of quaternization but is also strongly influenced by the chemical nature of the appended substituents, consistent with previous observations for N-functionalized tetrandrine derivatives [31,32,33]. Although N-quaternization changes the formal charge of the tetrandrine scaffold and may influence properties such as aqueous solubility, these physicochemical parameters were not experimentally quantified in the present work. Therefore, the observed differences in cytotoxicity cannot be attributed to a specific physicochemical change and should instead be interpreted as the combined consequence of the degree of substitution, the chemical nature of the appended groups, and the cellular context.

Based on their comparatively favorable viability profiles in ARPE-19 cells, **DBT**, **DeBT**, **DPBT**, **DIT**, **DAT** and **MU1NT** were selected for subsequent screening against human cancer cell lines. Importantly, their lower cytotoxicity toward ARPE-19 cells at this stage was not interpreted as evidence of reduced biological activity, but rather as a prerequisite for determining whether any of these derivatives could retain cytotoxic activity against cancer cells while exhibiting improved cellular selectivity. To establish a reference profile for subsequent comparison with the selected derivatives, the concentration-dependent cytotoxicity of the parent alkaloid **TET** was evaluated separately in ARPE-19 cells and in the five cancer cell lines, as described below.

#### 2.2.2. Concentration-Dependent Cytotoxicity of TET in Human Cell Lines

To establish a reference profile for the subsequent evaluation of the N-substituted derivatives, the concentration-dependent effect of the parent alkaloid **TET** was examined in non-cancerous ARPE-19 cells and in five human cancer cell lines: 22Rv1 prostate carcinoma, A549 lung adenocarcinoma, MDA-MB-231 triple-negative breast cancer, HCT-116 colorectal carcinoma, and HeLa cervical cancer cells. Following exposure to **TET** for 48 h, cell viability decreased in a concentration-dependent manner in all evaluated cell lines, allowing the corresponding IC_50_ values to be determined by dose–response curve fitting. A representative concentration–response profile for TET in 22Rv1 cells is shown in Figure S13.

As summarized in Table 1, the cytotoxic activity of **TET** varied among the evaluated cell lines. A549 cells were the most sensitive tumor model, with an IC_50_ of 4.23 ± 1.62 μM, followed by 22Rv1 cells, with an IC_50_ of 6.00 ± 1.96 μM. Higher IC_50_ values were obtained for HCT-116, MDA-MB-231, and HeLa cells, ranging from 15.88 to 17.09 μM. These differences indicate that the cellular response to **TET** is strongly dependent on the biological context, consistent with its context-dependent anticancer activity reported across different experimental models [3].

Importantly, **TET** also exhibited substantial cytotoxicity toward non-cancerous ARPE-19 cells, with an IC_50_ of 6.81 ± 1.79 μM. This value was comparable to that determined in 22Rv1 cells and was lower than those obtained in MDA-MB-231, HCT-116, and HeLa cells. Therefore, although **TET** reduced cell viability across the cancer cell line panel, its activity was not accompanied by consistent preferential cytotoxicity toward malignant cells. Representative bright-field microscopy images supported the MTT results, showing concentration-dependent reductions in cell confluence and alterations in cellular morphology in both cancer and non-cancerous cells. Although these qualitative observations do not establish a specific mechanism of cell death, they are consistent with the broad cytotoxic response of **TET** and its reported ability to affect multiple interconnected cellular processes, including energetic homeostasis, autophagic flux, oxidative and endoplasmic-reticulum stress, and intrinsic and extrinsic apoptotic signaling [10,11,12,13].

Taken together, these results demonstrate that **TET** possesses broad but poorly selective cytotoxic activity under the evaluated conditions. Its comparable effects in non-cancerous ARPE-19 cells and some of the most sensitive cancer cell lines, particularly 22Rv1, reveal an important limitation of the parent alkaloid. This experimental finding is consistent with the recognized pharmacological limitations of **TET**, including dose-dependent toxicity, poor aqueous solubility, limited oral bioavailability, and unfavorable pharmacokinetic properties, which continue to restrict its broader therapeutic development despite its well-established anticancer potential [3,7,8,17]. These limitations have motivated the structural modification of **TET** as a strategy to improve its physicochemical properties, cytotoxic potency, and selectivity toward cancer cells [8,17].

#### 2.2.3. Cytotoxicity Screening of Selected Tetrandrine Derivatives in Human Cancer Cell Lines

Following their initial evaluation in non-cancerous ARPE-19 cells, the derivatives with the most favorable viability profiles, **DBT**, **DeBT**, **DPBT**, **DIT**, **DAT** and **MU1NT** were evaluated against the five human cancer cell lines. Cells were exposed to the derivatives for 48 h at screening concentrations selected for each model: 95 μM for 22Rv1, 25 μM for A549, 15 μM for MDA-MB-231 and HCT-116, and 17 μM for HeLa cells, and doxorubicin was used as a positive control. Because the concentrations differed among cell lines, comparisons were made among compounds within each cellular model rather than directly across the different cell lines. Cell viability was determined using the MTT assay, and the results are summarized in Table 2.

The most pronounced cytotoxic responses were observed in 22Rv1 prostate carcinoma cells. At 95 μM, **DBT** and **MU1NT** reduced cell viability to 5.59 ± 1.96% and 4.72 ± 0.58%, respectively, values that were not significantly different from that obtained with doxorubicin at the same concentration. **DIT** and **DAT** also reduced 22Rv1 cell viability, although to a lesser extent, whereas **DeBT** and **DPBT** retained comparatively high viability percentages.

A different response pattern was observed in A549 lung adenocarcinoma cells. At 25 μM, **MU1NT** produced the greatest reduction in cell viability, reaching 13.34 ± 1.85%, comparable to the 16.12 ± 7.52% observed with doxorubicin. In contrast, the remaining derivatives maintained their percentage viability values above 50%, with **DBT**, **DeBT**, and **DPBT** exceeding 70%. Thus, within this screening experiment, **MU1NT** was the only derivative that exhibited pronounced cytotoxicity in both 22Rv1 and A549 cells.

The selected derivatives showed considerably lower cytotoxic activity in MDA-MB-231, HCT-116, and HeLa cells under the respective screening conditions. In MDA-MB-231 cells treated at 15 μM, all derivatives maintained cell viability above 84%, whereas doxorubicin reduced viability to 3.28 ± 1.00%. In HCT-116 cells, **DIT** produced the greatest reduction among the derivatives, although cell viability remained above 60%. Similarly, in HeLa cells, **DIT** produced the lowest viability value among the tested derivatives, but viability remained above 60%. These results indicate that none of the derivatives exhibited pronounced cytotoxicity against these three cell lines at the evaluated concentrations.

The markedly different responses observed across the tumor-cell panel demonstrate that the biological effects of the N-substituted derivatives depend on both the chemical nature of the appended groups and the cellular context. In particular, the contrasting behavior of **DBT** and **MU1NT** relative to structurally related derivatives indicates that cytotoxic activity cannot be predicted solely from the mono- or bis-substituted character of the compounds. This observation is consistent with previous studies from our group showing that relatively discrete changes in the N-attached substituents can substantially modify the molecular recognition and biological properties of the tetrandrine scaffold [29,31,32,33].

Taken together with their comparatively low cytotoxicity toward ARPE-19 cells during the initial screening, the pronounced effects of **DBT** and **MU1NT** in 22Rv1 cells, and of **MU1NT** in A549 cells, identified these compounds as the most promising candidates for subsequent dose–response studies. Their IC_50_ values were therefore determined in ARPE-19, 22Rv1, and A549 cells to quantitatively assess their cytotoxic potency and cellular selectivity, as described below.

Because these screening concentrations differed among the cellular models, the viability values were used only to compare derivatives within each cell line. In particular, the response observed in 22Rv1 cells at 95 μM cannot be compared directly with that observed in ARPE-19 cells at 25 μM or interpreted as evidence of cancer cell selectivity. Selectivity was subsequently evaluated using concentration–response assays and paired IC_50_ values obtained in the relevant cancer and non-cancerous cell models.

#### 2.2.4. IC_50_ Determination and Selectivity Analysis of DBT and MU1NT

Following the initial screening, concentration–response assays were performed for **DBT** and **MU1NT**, the two derivatives that showed the most promising combined profiles of low cytotoxicity toward non-cancerous ARPE-19 cells and cytotoxic activity against selected cancer cell lines. **DBT** was evaluated in ARPE-19 and 22Rv1 cells, whereas **MU1NT** was evaluated in ARPE-19, 22Rv1, and A549 cells because its initial screening profile indicated relevant cytotoxic activity in both tumor models. Cells were exposed to increasing concentrations of the compounds for 48 h, and the corresponding IC_50_ values were determined by dose–response curve fitting; a representative concentration–response profile is shown in Figure S14. The selectivity index (*SI*) was calculated as the ratio between the IC_50_ in non-cancerous ARPE-19 cells and the IC_50_ in each cancer cell line:

$$\mathrm{SI}=\frac{IC_{50\;}\mathrm{in ARPE}\text{-}19\;\mathrm{cells}}{IC_{50\;}\mathrm{in cancer cells}}$$

Accordingly, *SI* values greater than 1.0 indicate preferential cytotoxicity toward cancer cells, with progressively higher values reflecting greater discrimination between the cancer and non-cancerous models [39]. The resulting IC_50_ and *SI* values were compared with those obtained for the parent alkaloid **TET** and, in the 22Rv1 model, with docetaxel as a positive control (Table 3).

In ARPE-19 cells, **DBT** and **MU1NT** exhibited IC_50_ values of 45.44 ± 1.37 and 71.89 ± 3.33 μM, respectively, which were markedly higher than that obtained for **TET** (6.81 ± 1.79 μM). Thus, both derivatives were less cytotoxic against the non-cancerous reference model than the parent alkaloid, with **MU1NT** showing the lowest cytotoxicity among them. The IC_50_ of docetaxel in ARPE-19 cells was greater than 200 μM, indicating that 50% inhibition was not reached within the evaluated concentration range.

In 22Rv1 prostate carcinoma cells, **DBT** exhibited an IC_50_ of 9.89 ± 1.08 μM, whereas **MU1NT** showed a higher value of 28.00 ± 3.85 μM. Although **TET** was more potent, with an IC_50_ of 6.00 ± 1.96 μM, its comparable cytotoxicity toward ARPE-19 cells resulted in a low *SI* of 1.13. By contrast, **DBT** achieved an *SI* of 4.59, representing an approximately fourfold improvement over **TET** and the highest exactly determined *SI* among the tetrandrine-based compounds evaluated. **MU1NT** also showed improved discrimination relative to **TET**, although to a lesser extent, with an *SI* of 2.57. These results identify **DBT** as the derivative with the most favorable balance between cytotoxic potency in 22Rv1 cells and reduced cytotoxicity toward the non-cancerous ARPE-19 model.

Docetaxel, a clinically established treatment for advanced prostate cancer [40], exhibited an IC_50_ of 84.31 ± 4.48 μM in 22Rv1 cells under the present experimental conditions. Because its IC_50_ in ARPE-19 cells was greater than 200 μM, its *SI* could only be expressed as a lower bound of >2.37. Therefore, although the fully determined *SI* of **DBT** demonstrates marked and quantitatively resolved selectivity toward 22Rv1 cells, a definitive ranking of **DBT** and docetaxel based on *SI* is not possible because the exact selectivity of docetaxel was not determined within the evaluated concentration range.

The selectivity achieved by **DBT** is particularly noteworthy because this compound is not a naturally occurring alkaloid but a bis-N-substituted semisynthetic derivative generated through structural modification of **TET**. This N-functionalization strategy increased the *SI* in 22Rv1 cells from 1.13 for the parent alkaloid to 4.59 for **DBT**, representing an approximately fourfold improvement in cancer cell discrimination. The resulting selectivity is also relevant when considered in the context of other natural-product-derived compounds evaluated in 22Rv1 cells. For example, a synthetic 2-(chloroethylthio)-1,4-naphthoquinone derivative exhibited an IC_50_ of 0.14 ± 0.09 μM in 22Rv1 cells and an *SI* of 5.5 relative to non-cancerous PNT2 prostate cells, whereas the marine diterpene spongionellol A showed an IC_50_ of 2.64 ± 0.74 μM in 22Rv1 cells and an overall *SI* of 5.18 across panels of prostate cancer and non-cancerous cells [41,42]. N-Methylpretrichodermamide B, another marine natural product active in 22Rv1 cells, exhibited an IC_50_ of 3.02 ± 2.50 μM and an overall *SI* of 2.4 [43]. Although direct numerical comparisons should be interpreted cautiously because different non-cancerous reference models and *SI* calculation strategies were used, these studies place the *SI* of **DBT** within the range reported for other promising natural-product-derived candidates in prostate cancer models and support N-functionalization as an effective strategy for improving the selective cytotoxic profile of the tetrandrine scaffold.

In A549 lung adenocarcinoma cells, **MU1NT** exhibited an IC_50_ of 65.73 ± 4.46 μM, resulting in an *SI* of 1.09. **TET** showed a numerically higher *SI* of 1.61 because A549 cells were more sensitive to the parent alkaloid (IC_50_ = 4.23 ± 1.62 μM) than ARPE-19 cells. However, this ratio should not be interpreted as a favorable selectivity profile, because **TET** was highly cytotoxic toward both cell lines, with an IC_50_ of only 6.81 ± 1.79 μM in the non-cancerous ARPE-19 model. Conversely, although **MU1NT** was substantially less cytotoxic toward ARPE-19 cells, its similarly reduced potency in A549 cells resulted in an *SI* close to 1. Thus, neither compound achieved a favorable balance between cytotoxic potency and discrimination toward A549 cells under the evaluated conditions.

Overall, **DBT** showed the most favorable quantitatively determined selectivity profile, particularly in 22Rv1 cells, where it combined micromolar cytotoxic activity with a substantially reduced effect on the non-cancerous ARPE-19 model. Although **MU1NT** was less cytotoxic toward ARPE-19 cells, its lower potency in 22Rv1 and A549 cells resulted in lower selectivity indices. On this basis, **DBT** was selected as the lead derivative for further characterization of cell-death-associated morphological changes by fluorescence microscopy, cell death profiles by Annexin V-FITC/PI flow cytometry, and for molecular docking against apoptosis-related targets. Collectively, these findings support the prioritization of **DBT** as the most promising N-substituted tetrandrine derivative evaluated here and provide a strong rationale for its subsequent mechanistic investigation.

### 2.3. Fluorescence Microscopy Analysis of Nuclear and Cytoskeletal Alterations

To further examine the cellular alterations induced by the selected derivative, ARPE-19 and 22Rv1 cells were analyzed by fluorescence microscopy after 24 h of treatment with **TET**, **DBT**, or docetaxel. Each compound was evaluated at its corresponding IC_50_ value previously determined in 22Rv1 cells: 6.00 μM for **TET**, 9.89 μM for **DBT**, and 84.31 μM for docetaxel. Nuclear morphology and F-actin organization were visualized using DAPI and phalloidin staining, respectively. This paired analysis allowed the morphological effects of each treatment to be compared directly between the non-cancerous and prostate carcinoma cell models.

In ARPE-19 cells, treatment with **DBT** produced comparatively limited alterations in cell morphology, F-actin organization, and nuclear integrity relative to **TET** and docetaxel (Figure 3). In particular, the **DBT**-treated cells largely retained their spread morphology, organized actin cytoskeleton, and regular nuclear appearance. By contrast, **TET** caused a pronounced reduction in cell density, disruption of actin organization, cell shrinkage, and nuclear alterations, consistent with its substantial cytotoxicity and low selectivity toward the non-cancerous ARPE-19 model. These observations are also consistent with previous reports describing apoptosis-associated nuclear and cellular alterations following **TET** treatment in different cancer cell models [12,13]. Docetaxel also produced evident changes in cell morphology and cytoskeletal organization under the evaluated conditions (Figure 3).

In 22Rv1 prostate carcinoma cells, **DBT** induced marked morphological alterations, including cell shrinkage, loss or reduction in pseudopodial extensions, redistribution of F-actin toward the cell periphery, membrane blebbing, chromatin condensation, and nuclear fragmentation (Figure 4). These features are compatible with apoptosis-associated morphological remodeling, including actomyosin-dependent membrane blebbing and progressive nuclear disassembly [44,45,46]. Comparable treatment-associated changes detectable through combined DAPI and phalloidin staining have also been documented in 22Rv1 cells exposed to other bioactive preparations, supporting the usefulness of these complementary markers for visualizing nuclear and cytoskeletal damage in this cellular model [47]. Nevertheless, the present fluorescence observations are morphological and should not, by themselves, be interpreted as definitive evidence of a specific cell death pathway.

Treatment with **TET** also produced pronounced nuclear and cytoskeletal alterations in 22Rv1 cells, consistent with its cytotoxic activity in this cell line. However, similar damage was observed in ARPE-19 cells, reinforcing the absence of clear discrimination by the parent alkaloid between malignant and non-cancerous cells. Docetaxel induced substantial morphological alterations in 22Rv1 cells as well, providing a reference for the cellular damage produced by an established cytotoxic agent under the present experimental conditions.

Most importantly, the paired analysis of the two cell models revealed a clear qualitative contrast: **DBT** caused pronounced nuclear and cytoskeletal damage in 22Rv1 cells while largely preserving the morphology of ARPE-19 cells under the evaluated conditions. Conversely, **TET** produced substantial alterations in both cell types, in agreement with its poor discrimination between malignant and non-cancerous cells. This differential morphological response is consistent with the improved selectivity index of **DBT** and strengthens its selection for subsequent mechanistic evaluation.

### 2.4. Flow Cytometric Analysis of Cell Death Profiles in 22Rv1 Cells

Because the morphological alterations observed by fluorescence microscopy were compatible with, but not conclusive of, apoptosis, Annexin V-FITC/PI staining was used to characterize the cell death profiles induced by **TET** and **DBT** in 22Rv1 cells. Docetaxel was included as a reference cytotoxic agent. Cells were exposed for 24 h to each compound at the concentration corresponding to its 48 h IC_50_ value determined by the MTT assay. Representative cytograms are shown in Figure 5, and the distributions of viable, early apoptotic, late apoptotic, and Annexin V^−^/PI^+^ populations are summarized in Table 4. The representative untreated control contained 85.4% viable cells, whereas the DMSO-treated positive control showed the expected redistribution toward cell-death-associated populations. Annexin V/PI staining has also been used to characterize apoptosis induced by other bioactive compounds in the 22Rv1 cell model [41,48].

**TET** produced the largest apoptotic population among the evaluated treatments, reaching 86.30 ± 1.27% total apoptosis. The response was characterized by a predominance of early apoptotic cells and a comparatively small Annexin V^−^/PI^+^ population, indicating that **TET** predominantly increased Annexin V-positive populations rather than the population compatible with primary necrosis. These findings are consistent with previous studies showing that **TET** induces apoptosis in prostate cancer cells. Such studies have implicated caspase activation, PARP cleavage, modulation of BAX and Bcl-2, inhibition of Akt signaling, and effects on death-receptor-associated pathways [11,14,49]. However, the present Annexin V-FITC/PI assay does not establish which of these molecular events contributed to the response observed in 22Rv1 cells.

**DBT** produced a smaller but clearly detectable apoptotic population, with total apoptosis reaching 34.77 ± 5.13%. Early apoptosis predominated over late apoptosis, and Annexin V-positive cells were more abundant than the Annexin V^−^/PI^+^ population. Thus, the reduction in viability associated with **DBT** was accompanied predominantly by phosphatidylserine externalization, providing direct experimental evidence of an apoptosis-associated phenotype in 22Rv1 cells under the evaluated conditions. Docetaxel produced 32.17 ± 3.07% total apoptosis, and its overall cell-population distribution was descriptively similar to that observed with **DBT**. Previous work has shown a comparatively limited apoptotic response to docetaxel in 22Rv1 cells relative to castration-sensitive prostate cancer models [50]. Moreover, because flow cytometry was conducted only in 22Rv1 cells, these results do not independently demonstrate differential apoptotic selectivity between 22Rv1 and ARPE-19 cells. Overall, the flow cytometry results confirm that both **TET** and **DBT** can induce an apoptosis-associated response in 22Rv1 cells, while also revealing a markedly greater response to **TET** at 24 h. These differences describe the cellular responses observed at the respective treatment concentrations but should not be interpreted as a direct comparison of intrinsic potency or as evidence of a specific molecular target.

### 2.5. Molecular Docking Analysis of TET and DBT Against Apoptosis-Related Targets

Molecular docking was performed to explore whether the structural modification introduced in **DBT** could alter its predicted interactions with proteins associated with apoptotic signaling. DR4 and DR5 were selected as representative components of the TRAIL-mediated extrinsic pathway, whereas Bcl-2, Bcl-XL, and BAX were included as regulators of the mitochondrial apoptotic pathway. The selection of these proteins was supported by previous evidence that **TET** can influence death receptor signaling and modulate apoptosis-associated processes involving Bcl-2-family proteins in cancer cells [7,11,14,34]. The analysis was intended as a hypothesis-generating comparison of the interaction profiles predicted for **TET** and **DBT**, rather than as evidence establishing these proteins as direct intracellular targets.

The predicted binding energies obtained for the top-ranked docking poses are summarized in Table 5. Both compounds produced favorable docking scores across the evaluated protein panel, although their relative profiles differed. For the death receptors, **TET** showed predicted binding energies of −12.10 kcal/mol for DR5 and −12.20 kcal/mol for DR4, whereas the corresponding values for **DBT** were −11.30 and −11.20 kcal/mol, respectively. Thus, **TET** produced more favorable scores for both death receptors, although **DBT** retained favorable predicted interactions with these proteins. Inspection of the selected poses indicated that the **TET** complexes were stabilized predominantly by hydrophobic and π-alkyl contacts, accompanied by a smaller contribution from polar interactions (Figure 6). By comparison, the dicationic **DBT** derivative displayed a greater electrostatic component, including attractive-charge and π-cation or π-anion contacts, together with hydrophobic interactions (Figure 7).

A different comparative pattern was observed for the Bcl-2-family proteins. **DBT** produced more favorable predicted binding energies than **TET** for Bcl-2 (−10.40 versus −9.00 kcal/mol), Bcl-XL (−10.60 versus −9.40 kcal/mol), and BAX (−9.80 versus −9.00 kcal/mol). The selected **DBT** poses showed electrostatic contacts with acidic residues, together with aromatic and hydrophobic interactions (Figure 7), whereas the corresponding **TET** poses appeared to depend more strongly on hydrophobic and π-type contacts (Figure 6). The greater electrostatic contribution observed for **DBT** is chemically consistent with its formal dicationic state and the substituents introduced through N-functionalization. Nevertheless, docking scores should be interpreted as relative predictions generated by the scoring function and not as experimentally determined binding free energies.

Collectively, the docking analysis revealed that N-functionalization changed the predicted interaction profile of the tetrandrine scaffold. **TET** showed the most favorable scores toward DR4 and DR5, whereas **DBT** showed comparatively improved scores toward Bcl-2, Bcl-XL, and BAX while retaining favorable predicted interactions with both death receptors. This distribution suggests that the structural modification in **DBT** may modify its molecular recognition properties across apoptosis-related proteins rather than uniformly increasing its predicted binding to every evaluated target. However, because the functional consequences of ligand binding cannot be inferred from docking scores alone, the predicted interaction of **DBT** with BAX cannot be interpreted as activation of this pro-apoptotic protein, nor can its predicted interactions with Bcl-2 or Bcl-XL be assumed to produce inhibition. These computational results provide a hypothesis-generating framework that is compatible with the experimental profile of **DBT**, including its improved selectivity toward 22Rv1 cells and the nuclear and cytoskeletal alterations observed by fluorescence microscopy. In particular, its predicted interactions with both death receptors and mitochondrial apoptosis-related proteins identify potential molecular nodes for subsequent investigation. Nevertheless, the docking results do not demonstrate that these proteins mediate the cellular response to **DBT**, and their correspondence with the fluorescence observations should be regarded as convergent but indirect evidence. Target-engagement studies and biochemical analyses of apoptosis-related signaling, including DR4/DR5 expression, regulation of Bcl-2-family proteins, caspase activation, and PARP cleavage, will be required to determine whether the extrinsic or mitochondrial apoptotic pathway contributes to the cytotoxic effect of **DBT** in 22Rv1 cells.

Overall, N-functionalization altered the predicted interaction profile of the tetrandrine scaffold rather than uniformly improving its docking scores across all evaluated targets. **TET** produced more favorable scores toward DR4 and DR5, whereas **DBT** showed comparatively more favorable scores toward Bcl-2, Bcl-XL, and BAX. Nevertheless, these blind docking calculations remain exploratory: docking scores do not establish cellular target engagement or indicate whether ligand binding would activate or inhibit the evaluated proteins. To assess the ability of the docking procedure to reproduce an experimentally determined binding mode, a complementary site-specific validation was performed for Bcl-2 using the co-crystallized inhibitor venetoclax.

The reported values correspond to the top-ranked docking poses with the most favorable predicted binding energies (Δ*G*) obtained using AutoDock Vina, version 1.2.7. More negative scores indicate a more favorable predicted interaction within the applied docking protocol. The corresponding binding poses and protein–ligand interaction maps are presented in Figure 6 and Figure 7. These blind docking calculations used PDB entries 1D0G (DR5), 5CIR (DR4), 6O0K (Bcl-2), 1R2D (Bcl-XL) and 6EB6 (BAX). They are not interchangeable with the targeted Bcl-2 values in Table 6, which were obtained on a different search space.

#### 2.5.1. Reference-Site Docking and Redocking Validation

A targeted docking protocol was established for the venetoclax-binding site of Bcl-2 using the crystal structure 6O0K [51]. Redocking of the co-crystallized inhibitor recovered its experimental conformation as the top-ranked pose, with a symmetry-corrected heavy-atom RMSD of 0.55 Å and a predicted binding score of −12.2 kcal/mol (Table 6 and Figure S15). This value was below the prespecified 2.0 Å criterion, supporting the ability of the site-specific protocol to reproduce the reference binding mode. When docked into the same validated site, **TET** and **DBT** produced scores of −7.8 and −8.3 kcal/mol, respectively, both less favorable than that obtained for venetoclax. The difference of 0.5 kcal/mol between **TET** and **DBT** is too small to account for their distinct cellular selectivity profiles and should not be interpreted as evidence of differential Bcl-2 inhibition. Because the targeted calculations used a different search space and receptor-preparation procedure, these scores are not directly interchangeable with the blind docking values reported in Table 5. Equivalent redocking validation was not possible for DR4, DR5, Bcl-XL, or BAX using the selected structures because they lacked an appropriate co-crystallized small-molecule reference ligand; consequently, their docking results remain exploratory.

#### 2.5.2. Cell Line RNA Expression Context

Baseline RNA expression data were examined to determine whether the apoptosis-related components selected for docking were transcriptionally represented in the cellular models used in the study. Human Protein Atlas data showed detectable transcripts for TNFRSF10A, TNFRSF10B, BCL2, BCL2L1, and BAX in all five cancer cell lines [52]. In 22Rv1 cells, the corresponding nTPM values were 13.3, 29.7, 3.2, 20.3, and 59.0, respectively. Across the cancer cell panel, BCL2 showed comparatively low transcript abundance, whereas BCL2L1 and BAX were generally more abundant (Table S1).

Because ARPE-19 was not represented in the Human Protein Atlas cell line dataset, its baseline expression was examined separately using four untreated or mock-control samples from GEO series GSE159235 [53]. All five genes were detected in these control samples (Table S2). However, these CPM values were obtained using an independent dataset and processing pipeline and were therefore not compared quantitatively with the HPA nTPM values.

Publicly available proteomic resources were also examined; however, they did not provide a methodologically comparable dataset covering both the cancer cell panel and the non-cancerous ARPE-19 model [54,55]. Therefore, the expression analysis establishes the transcriptional presence of the selected pathway components but does not demonstrate protein abundance, ligand binding, or treatment-induced pathway engagement.

## 3. Materials and Methods

### 3.1. Reagents, Synthesis, and Characterization

All reagents and solvents used for chemical synthesis were purchased from Sigma-Aldrich (St. Louis, MO, USA) and used as received without further purification. The previously reported tetrandrine derivatives **DBT**, **DeBT**, **DIT**, **DNT**, **DQT**, **DAcT**, and **DAT**, as well as the monosubstituted derivatives **MAnT** and **MAcT**, were prepared according to literature procedures [24,27,29,30,31,33]. The previously reported chloroethylurea intermediate **U1N**, used as the synthetic precursor for the preparation of **MU1NT**, was prepared following a procedure similar to that described by Johnston et al. (1966) [38]. The synthetic procedures and characterization data for the new tetrandrine derivatives **MU1NT** and **DPBT** are described below. For completeness, the preparation and characterization of the previously reported intermediate **U1N** are also included.

### 3.2. Instrumentation

Nuclear magnetic resonance (NMR) spectra were recorded on a Bruker Avance III 400 spectrometer (Bruker BioSpin GmbH, Rheinstetten, Germany). Decomposition points were determined using a Büchi B-545 melting point apparatus (BÜCHI Labortechnik AG, Flawil, Switzerland). Infrared (IR) spectra were recorded on a PerkinElmer FRONTIER FTIR spectrophotometer (PerkinElmer, Shelton, CT, USA) using an attenuated total reflectance (ATR) accessory. Mass spectra were acquired on an Agilent Technologies 6100 LC/MS system (Agilent Technologies, Santa Clara, CA, USA) operating in positive electrospray ionization (ESI+). Bright-field and fluorescence images were acquired using a Leica DMi8 inverted epifluorescence microscope (Leica Microsystems, Wetzlar, Germany) equipped with a 20× objective. Flow cytometry experiments were performed using a FACSCanto II flow cytometer (BD Biosciences, San Jose, CA, USA).

### 3.3. General Procedure for the Preparation of Mono- and Bis-N-Substituted Tetrandrine Derivatives

Tetrandrine was dissolved in acetone, and the corresponding alkylating agent was added. The reaction mixture was heated under reflux for the appropriate reaction time while monitoring the reaction progress by TLC. Upon completion of the reaction, the solvent was removed under reduced pressure, and the product was precipitated by the addition of ethyl acetate. The precipitate was collected by vacuum filtration, washed successively with ethyl acetate to remove residual impurities, and dried in a vacuum oven to afford the corresponding N-substituted tetrandrine derivative.

#### 3.3.1. Synthesis of MU1NT

**MU1NT** was prepared according to the general procedure using 0.2 g of tetrandrine (Supelco, Sigma-Aldrich, St. Louis, MO, USA; Cat. No. T2695) (0.29 mmol) and 0.08 g of the chloroethylurea derivative **U1N** (0.32 mmol) as the alkylating agent. The reaction mixture was refluxed for 48 h to afford **MU1NT** as a white solid (0.104 g, 40%). MP 173–175 °C. ^1^H NMR (400 MHz, DMSO-d_6_, δ ppm) partial signal assignment was supported by two-dimensional NMR techniques: Signals for -N^+^-CH_3_ and -O-CH_3_ were found at 2.89 (s, 3H), 3.33 (s, 3H), 3.34 (s, 3H), 3.51 (s, 3H), 3.62 (s, 3H), 3.81 (s, 3H). Signals corresponding to the methylene -N^+^-(CH_2_)_2_-R and methine -N^+^-CH-R protons appeared between δ 3.6 and 5.2 ppm. The ten aromatic protons of the tetrandrine skeleton resonated at δ 6.11 (s, 1H), 6.36 (m, 3H), 6.74 (m, 2H), 6.86 (s, 1H), 6.94 (d, J = 8.2 Hz, 1H), 7.16 (dd, J = 8.3, 2.6 Hz, 1H), and 7.39 (t, J = 7.9 Hz, 1H). The aromatic protons of the naphthyl group resonated between δ 7.43 and 8.31 ppm. The NH proton attached to the naphthyl-substituted nitrogen of the urea group resonated at δ 9.17 ppm. ^13^C NMR (100 MHz, DMSO-d_6_, δ, ppm): 156.36 (urea C=O); 154.22–107.14 (aromatic carbons of the tetrandrine skeleton and the pendant naphthyl group); 69.80–21.25 (-O-CH_3_, -N^+^-CH_3_, -N^+^-CHR, and N^+^–(CH_2_)_2_-R carbons of the pendant arm). FTIR-ATR (cm^−1^): 3377 (N–H stretching), 2929 and 2834 (aliphatic C–H stretching), 1608 (urea C=O stretching), 1582 (aromatic ring C=C stretching), 1503 (N–H bending coupled with aromatic ring stretching), 1446 (CH_2_ and CH_3_ bending), 1263 and 1227 (C–O–C and C–N stretching), 1119 and 1018 (methoxy C–O and ether C–O–C stretching), and 840 (aromatic C–H out-of-plane bending). MS-ESI(+) *m*/*z*: 835.0 ([M-Cl]^+^, 100%).

#### 3.3.2. Synthesis of DPBT

**DPBT** was prepared according to the general procedure using 0.2 g of tetrandrine (0.29 mmol) and 0.15 g of 1-bromo-3-phenylpropane (0.725 mmol) as the alkylating agent. The reaction mixture was refluxed for 48 h to afford **DPBT** as a white solid (0.192 g, 65%). MP 238–241 °C. ^1^H NMR (400 MHz, DMSO-d_6_, δ ppm) partial signal assignment was supported by two-dimensional NMR techniques: Signals for -N^+^-CH_3_ and -O-CH_3_ were found at 2.57 (s, 3H), 3.21 (s, 3H), 3.34 (s, 3H), 3.48 (s, 3H), 3.78 (s, 3H), 3.80 (s, 3H). Signals corresponding to the -N^+^-(CH_2_)_3_-R methylene protons appeared between δ 3.63 and 5.1 ppm. The chemical shifts in nine of the ten aromatic protons of the tetrandrine skeleton were observed at 7.13 (s, 1H), 7.10 (dd, J = 8.4, 2.0 Hz, 1H), 6.97 (d, J = 8.4 Hz, 1H), 6.93 (dd, J = 8.3, 2.6 Hz, 1H), 6.82 (s, 1H), 6.71 (dd, J = 8.1, 2.6 Hz, 1H), 6.57 (dd, J = 8.2, 2.2 Hz, 1H), 6.41 (d, J = 2.1 Hz, 1H), 6.06 (s, 1H). The remaining aromatic proton signals, corresponding to one proton of the tetrandrine core and the protons of the two pendant phenyl groups, appeared as overlapping multiplets between 7.3 and 7.6 ppm, preventing their individual assignment. ^13^C NMR (100 MHz, DMSO-d_6_, δ, ppm): 154.88–100.15 (aromatic carbons of the tetrandrine skeleton and the two pendant phenyl groups); 67.83–23.44 (-O-CH_3_, -N^+^-CH_3_, -N^+^-CHR, and carbons of the two N^+^–(CH_2_)_3_–Ph arms). MS-ESI(+) *m*/*z*: 941.8 ([M-Br]^+^, 0.99%), 430.2 ([M-2Br]^2+^, 100%).

#### 3.3.3. Synthesis of U1N (Previously Reported)

Compound **U1N** was prepared following a procedure similar to that described by Johnston et al. [38]. Briefly, 1-naphthylamine (0.500 g, 3.39 mmol) was dissolved in anhydrous dichloromethane (35 mL) under a nitrogen atmosphere. A solution of 2-chloroethyl isocyanate (0.335 g, 3.08 mmol) in anhydrous dichloromethane (5 mL) was then added, and the reaction mixture was stirred at room temperature overnight. Upon standing overnight at room temperature, **U1N** precipitated as a white solid. The precipitate was collected by vacuum filtration, washed successively with dichloromethane to remove residual impurities, and dried in a vacuum oven to afford **U1N** as a white solid (0.71 g, 90%). MP 151–153 °C. ^1^H NMR (400 MHz, DMSO-d_6_, δ ppm): Signals assigned to the two urea NH protons were observed at 8.69 (s, 1H) and 6.89 (t, J = 5.6 Hz, 1H). The seven aromatic protons of the 1-naphthyl group resonated between 7.43 and 8.10, with characteristic resonances at 8.10 (d, J = 8.2 Hz, 1H), 7.99 (d, J = 7.2 Hz, 1H), 7.90 (m, 1H), 7.59–7.50 (m, 3H), 7.43 (t, J = 7.9 Hz, 1H). Signals corresponding to the 2-chloroethyl moiety were observed at 3.72 (t, J = 6.1 Hz, 2H), 3.49 (m, 2H). ^13^C NMR (100 MHz, DMSO-d_6_, δ, ppm): 155.99 (urea C=O), 135.40, 134.19, 128.32, 126.37, 126.25, 126.08, 125.93, 122.76, 121.90, 117.16 (naphthyl carbons), 45.10 (CH_2_Cl), and 41.83 (NHCH_2_). FTIR-ATR (cm^−1^): 3360 and 3305 (N–H stretching), 2929 and 2835 (aliphatic C–H stretching), 1610 (urea C=O stretching), 1582 (aromatic ring C=C stretching), 1502 (N–H bending coupled with aromatic ring stretching), 1448 (CH_2_ bending and aromatic ring vibration), 1259 and 1228 (C–N stretching), and 840 and 754 (aromatic C–H out-of-plane bending). ESI-MS (ESI+) *m*/*z*: 271.4 [M + Na]^+^, 519.6 [2M + Na]^+^. Anal. Calcd for C_13_H_13_ClN_2_O: C, 62.78; H, 5.27; N, 11.26. Found: C, 62.58; H, 5.21; N, 11.25. The spectroscopic data were consistent with those previously reported [38].

### 3.4. Cell Culture and Sample Treatment

Human cancer cell lines, including prostate adenocarcinoma (22Rv1, ATCC CRL-2505), lung adenocarcinoma (A549, ATCC CCL-185), triple-negative breast adenocarcinoma (MDA-MB-231, ATCC HTB-26), colorectal carcinoma (HCT-116, ATCC CCL-247), and cervical adenocarcinoma (HeLa, ATCC CCL-2), together with the non-tumor retinal pigment epithelial cell line ARPE-19 (ATCC CRL-2302), all obtained from the American Type Culture Collection (ATCC, Manassas, VA, USA), were used to evaluate the cytotoxic activity of **TET** and its derivatives. The 22Rv1 cell line was maintained in Roswell Park Memorial Institute medium (RPMI-1640; Sigma-Aldrich, St. Louis, MO, USA), whereas the remaining cell lines were cultured in Dulbecco’s Modified Eagle Medium (DMEM; Sigma-Aldrich, St. Louis, MO, USA). Culture media were supplemented with fetal bovine serum (FBS; Gibco, Thermo Fisher Scientific, Waltham, MA, USA; Cat. No. 16000044), gentamicin, amphotericin B, and ampicillin/streptomycin (all from Sigma-Aldrich, St. Louis, MO, USA), and cells were maintained at 37 °C in a humidified atmosphere containing 5% CO_2_.

Stock solutions of **TET** and its derivatives (**DBT**, **DeBT**, **DPBT**, **DIT**, **DNT**, **DQT**, **DAcT**, **DAT**, and **MU1NT**) were prepared in dimethyl sulfoxide (DMSO; cell culture grade; Sigma-Aldrich, St. Louis, MO, USA) and diluted with the corresponding culture medium immediately before use to obtain the desired treatment concentrations. Cells treated with the corresponding concentration of DMSO served as the vehicle control throughout all experiments.

### 3.5. MTT Cell Viability Assay

Cell viability was determined using the MTT reduction assay with minor modifications of the method originally described by Mosmann [56]. Human cells were seeded into 96-well microplates at a density of 1 × 10^4^ cells per well and allowed to attach for 24 h under standard culture conditions. According to the experimental design, cells were treated for 48 h with either a single concentration or a range of concentrations of **TET** or its derivatives. In the initial screening assays, **TET** and doxorubicin were included as reference compounds. For the subsequent concentration–response studies in 22Rv1 cells, dose–response curves were generated for **TET**, **DBT**, **MU1NT**, and docetaxel, whereas doxorubicin was included as a positive cytotoxic control at a single concentration in each experiment. Cells treated with DMSO (cell culture grade; Sigma-Aldrich, St. Louis, MO, USA) alone served as the vehicle control.

After treatment, 10 μL of MTT solution (5 mg/mL; Sigma-Aldrich, St. Louis, MO, USA) was added to each well, and the plates were incubated for an additional 4 h at 37 °C. The culture medium was then removed, and the resulting formazan crystals were dissolved in 50 μL of DMSO. Absorbance was measured at 570 nm using 630 nm as the reference wavelength with an 800 TS microplate reader (BioTek Instruments, Winooski, VT, USA). Cell viability was expressed as a percentage relative to the vehicle control. Concentration–response curves were generated for **TET**, **DBT**, **MU1NT**, and docetaxel, and IC_50_ values were calculated using NCSS 2025 statistical software (NCSS, LLC, Kaysville, UT, USA).

### 3.6. DAPI and Phalloidin Fluorescence Staining

Fluorescence staining was performed following the method described by Van Vuuren et al. (2019) [57], with minor modifications. According to the experimental design, 22Rv1 or ARPE-19 cells were seeded into 96-well microplates at a density of 1 × 10^4^ cells per well and allowed to attach for 24 h under standard culture conditions. Cells were then treated with **TET**, **DBT**, or docetaxel at the corresponding IC_50_ concentration for 24 h. After treatment, the cells were fixed with 3.7% formaldehyde in phosphate-buffered saline (PBS) and subsequently permeabilized with 0.2% Triton X-100 in PBS for 15 min.

F-actin was stained with TRITC-phalloidin (50 μg/mL), whereas nuclei were counterstained with DAPI dilactate (1.5 μg/mL). All commercial reagents used in this procedure were obtained from Sigma-Aldrich (St. Louis, MO, USA).

### 3.7. Flow Cytometric Analysis of Cell Death

Cell death was evaluated by Annexin V-FITC/propidium iodide (PI) staining using the Annexin V-FITC Apoptosis Detection Kit (Invitrogen™, Thermo Fisher Scientific, Waltham, MA, USA; Cat. No. BMS500FI-100), according to the manufacturer’s instructions with modifications. Briefly, 1 × 10^5^ 22Rv1 cells were seeded in 12-well plates containing 500 µL of FBS-supplemented RPMI medium per well and incubated for 24 h. Subsequently, the medium was replaced with fresh FBS-supplemented RPMI containing **TET**, **DBT**, or docetaxel at their respective IC_50_ concentrations, previously determined by the MTT assay after 48 h of exposure, and cells were incubated for an additional 24 h. Untreated cells were used as the negative control, whereas cells exposed to 20% DMSO for 24 h were used as a positive cell death control and to prepare the single-stained compensation controls.

After treatment, the culture medium containing floating cells was collected. The adherent cells were washed with 1× phosphate-buffered saline (PBS), detached using trypsin, and combined with the corresponding floating-cell fraction; PBS and trypsin were obtained from Sigma-Aldrich (St. Louis, MO, USA). The resulting cell suspensions were centrifuged at 250× *g* for 5 min at 25 °C, and the supernatant was discarded. The cell pellet was resuspended in 195 µL of binding buffer, followed by the addition of 5 µL of Annexin V-FITC. Samples were incubated for 15 min at room temperature in the dark and subsequently centrifuged at 250× *g* for 5 min. After removal of the supernatant, cells were washed with binding buffer and centrifuged again under the same conditions. The resulting cell pellet was resuspended in 190 µL of binding buffer, and 10 µL of PI (20 µg/mL) was added. Samples were incubated for an additional 15 min at room temperature in the dark.

Untreated unstained cells were used to identify the cell population, whereas cells exposed to 20% DMSO and stained individually with Annexin V-FITC or PI were used to perform fluorescence compensation. DMSO-treated cells stained with both Annexin V-FITC and PI were included as the positive cell death control, and untreated double-stained cells served as the negative control.

Finally, the cell suspensions were filtered, transferred to flow cytometry tubes, and analyzed using a FACSCanto II™ flow cytometer (BD Biosciences, San Jose, CA, USA). Approximately 12,000–15,000 events were acquired per sample. A sequential gating strategy based on forward- and side-scatter parameters was used to select the cellular population and exclude debris and cell aggregates; the resulting P4 population was used for Annexin V-FITC/PI analysis. Data acquisition and analysis were performed using BD FACSDiva software, version 6.1.2 (BD Biosciences, San Jose, CA, USA). Cell populations were classified according to Annexin V-FITC and PI fluorescence as viable (Annexin V^−^/PI^−^), early apoptotic (Annexin V^+^/PI^−^), late apoptotic (Annexin V^+^/PI^+^), or primarily necrotic (Annexin V^−^/PI^+^) cells. The assay was conducted as a single experiment with technical repetitions.

### 3.8. Molecular Docking Studies

The crystal structure of **TET** was obtained from previously reported single-crystal X-ray diffraction studies by Gilmore et al. (1976) and Zhou et al. (2014) [58,59]. The geometry of **DBT** corresponded to the DFT-optimized structure previously reported by González-Martínez et al. (2022) [31], using crystallographic coordinates as the starting geometry. Both structures were used as ligands for molecular docking calculations. The crystal structures of the apoptosis-related proteins death receptor 5 (DR5, PDB ID: 1D0G), death receptor 4 (DR4, PDB ID: 5CIR), B-cell lymphoma 2 (Bcl-2, PDB ID: 6O0K), B-cell lymphoma-extra large (Bcl-XL, PDB ID: 1R2D), and Bcl-2-associated X protein (BAX, PDB ID: 6EB6) were retrieved from the Protein Data Bank (PDB).

The receptor structures were prepared using AutoDockTools (MGLTools), version 1.5.6 (The Scripps Research Institute, La Jolla, CA, USA) by removing crystallographic water molecules and other non-essential molecules, adding polar hydrogen atoms, assigning Kollman charges, and converting the structures into PDBQT format. Ligand structures were also prepared using AutoDockTools by assigning Gasteiger charges, defining rotatable bonds, and converting them into PDBQT files.

Molecular docking calculations were performed using AutoDock Vina version 1.2.7 (The Scripps Research Institute, La Jolla, CA, USA) [60]. Blind docking was carried out by defining a search grid covering the entire protein structure to identify potential binding sites without imposing prior assumptions regarding the binding pocket. Default docking parameters were employed, considering rigid receptor structures and fully flexible ligands. For each protein–ligand complex, the binding pose with the lowest predicted binding energy was selected for further analysis.

Protein–ligand interactions, including hydrogen bonds, hydrophobic contacts, π–π interactions, and other non-covalent interactions, were analyzed and visualized using BIOVIA Discovery Studio Visualizer, version 2023 (Dassault Systèmes, San Diego, CA, USA) [61].

#### 3.8.1. Targeted Bcl-2 Docking and Redocking Validation

An additional targeted docking analysis was performed using the crystal structure of human Bcl-2 in complex with venetoclax (**PDB ID: 6O0K**; resolution, 1.62 Å) [51]. Chain A was retained, and alternate-location conformer A was selected for venetoclax. Crystallographic water molecules and non-essential heteroatoms were removed, and the receptor was protonated at pH 7.4 using PDB2PQR 3.7.1 (APBS–PDB2PQR project) with the AMBER force field and PROPKA, version 3.0 (Jensen Group, Department of Chemistry, University of Copenhagen, Copenhagen, Denmark) [62]. Numerical array calculations, binding score matrices, and data processing were carried out using NumPy 2.4.6 (NumPy open-source project) [63], whereas graphical plots and affinity profiles were constructed using Matplotlib 3.10.8 (Matplotlib open-source project) [64]. Receptor and ligand PDBQT files were generated using Meeko 0.8.0 (Forli Lab, Scripps Research, La Jolla, CA, USA), with Gasteiger charges. The macrocyclic frameworks of **TET** and **DBT** were kept intact during ligand preparation.

The docking box was centered on the co-crystallized venetoclax-binding site at *x* = −14.226, *y* = 1.146, and *z* = −10.800 Å, with dimensions of 20.000 × 21.916 × 28.898 Å. Calculations were performed using AutoDock Vina 1.2.7 with an exhaustiveness value of 32, 20 requested binding modes, an energy range of 6 kcal/mol, four CPUs, and a random seed of 20260830. The same receptor, grid, and search settings were used for venetoclax redocking and the docking of **TET** and **DBT**.

Redocking accuracy was evaluated by comparing the predicted and crystallographic coordinates of venetoclax. Symmetry-corrected RMSD was calculated over ligand heavy atoms using RDKit 2026.03.5 (RDKit open-source project). The coordinate sets were compared directly in the receptor reference frame without post-docking fitting of the predicted pose. A heavy-atom RMSD of ≤2.0 Å for the top-ranked pose was prespecified as the criterion for successful redocking.

#### 3.8.2. Public Cell Line RNA Expression Analysis and Proteomic Data Screening

Baseline RNA expression data for TNFRSF10A, TNFRSF10B, BCL2, BCL2L1, and BAX were obtained from the Human Protein Atlas (HPA) cell line dataset, version 25.1 (Ensembl 109) [52]. The five cancer cell lines evaluated experimentally were matched to the HPA labels 22Rv1, A-549, MDA-MB-231, HCT 116, and HeLa, and normalized transcript abundance was reported as nTPM. Because ARPE-19 was not represented in the HPA cell line dataset, its baseline expression was assessed separately using raw-count data from four untreated or mock-treated control samples from GEO series GSE159235 [53]. Raw gene counts were converted to counts per million (CPM) by dividing each gene count by the sum of all gene counts within the corresponding sample and multiplying by 10^6^. HPA nTPM and GEO CPM values were not compared quantitatively because they were obtained from independent datasets and processing pipelines.

Two public proteomic resources, including a pan-cancer proteomic map, were also screened for the abundance of the proteins encoded by the five selected genes in the experimental cell models [54,55]. Protein-abundance data were not incorporated into Tables S1 and S2 because neither resource included ARPE-19 and, therefore, they could not provide a complete protein-level comparison across all experimental cell models. Moreover, the reported abundance values were generated using independent normalization procedures.

### 3.9. Statistical Analysis

Data from the MTT assays are presented as the mean ± standard error of the mean (SEM) from three independent experiments, each performed in triplicate. Half-maximal inhibitory concentration (IC_50_) values were calculated using NCSS 2025 Statistical Software (version 2025, NCSS, LLC, Kaysville, UT, USA) [65]. Statistical comparisons among experimental groups in the MTT assays were performed using one-way analysis of variance (ANOVA), followed by the Tukey–Kramer multiple-comparisons test. Differences were considered statistically significant at *p* < 0.05.

Flow cytometry data were analyzed descriptively and are presented as the mean ± standard deviation (SD) of the available technical repetitions from a single experiment (**TET**, *n* = 2; **DBT** and docetaxel, *n* = 3). Total apoptosis was calculated separately for each technical repetition as the sum of the early- and late apoptotic populations before calculation of the mean and SD. No inferential statistical comparisons were performed for the flow cytometry data.

## 4. Conclusions

The present study shows that N-functionalization can substantially modify the biological profile of the tetrandrine scaffold and, for selected derivatives, enhance selectivity between malignant and non-cancerous cells. The initial evaluation in ARPE-19 cells served as a preliminary selection step before testing selected derivatives across a heterogeneous panel of human cancer cell lines. In contrast, the parent alkaloid **TET** showed poor selectivity between 22Rv1 and ARPE-19 cells.

Among the evaluated derivatives, **DBT** displayed the most favorable balance between cytotoxic potency and selectivity in the 22Rv1 model, achieving an *SI* of 4.59 compared with 1.13 for **TET**. **MU1NT** also showed improved discrimination toward 22Rv1 cells, although it was less potent than **DBT**. These findings indicate that the biological consequences of N-substitution depend on both the incorporated structural motif and the cellular context.

Fluorescence microscopy supported the differential cellular responses identified by the viability assays, whereas Annexin V-FITC/PI flow cytometry demonstrated an apoptosis-associated response in **DBT**-treated 22Rv1 cells. However, these findings neither identify the molecular pathway involved nor independently demonstrate differential apoptotic selectivity between 22Rv1 and ARPE-19 cells.

Molecular docking was therefore interpreted as hypothesis-generating rather than as evidence of a defined mechanism. Targeted redocking of co-crystallized venetoclax validated the Bcl-2 site-specific protocol (RMSD = 0.55 Å); however, the small score difference between **TET** and **DBT** did not account for their distinct cellular selectivity profiles. Public RNA expression data established the transcriptional presence of the selected apoptosis-related genes in the cellular models, although neither transcript abundance nor docking predictions demonstrate protein-level target engagement. Overall, **DBT** represents the most promising derivative for further mechanistic evaluation, including studies in tissue-matched non-cancerous cells and in vivo prostate cancer models.

## Figures and Tables

**Figure 1:**
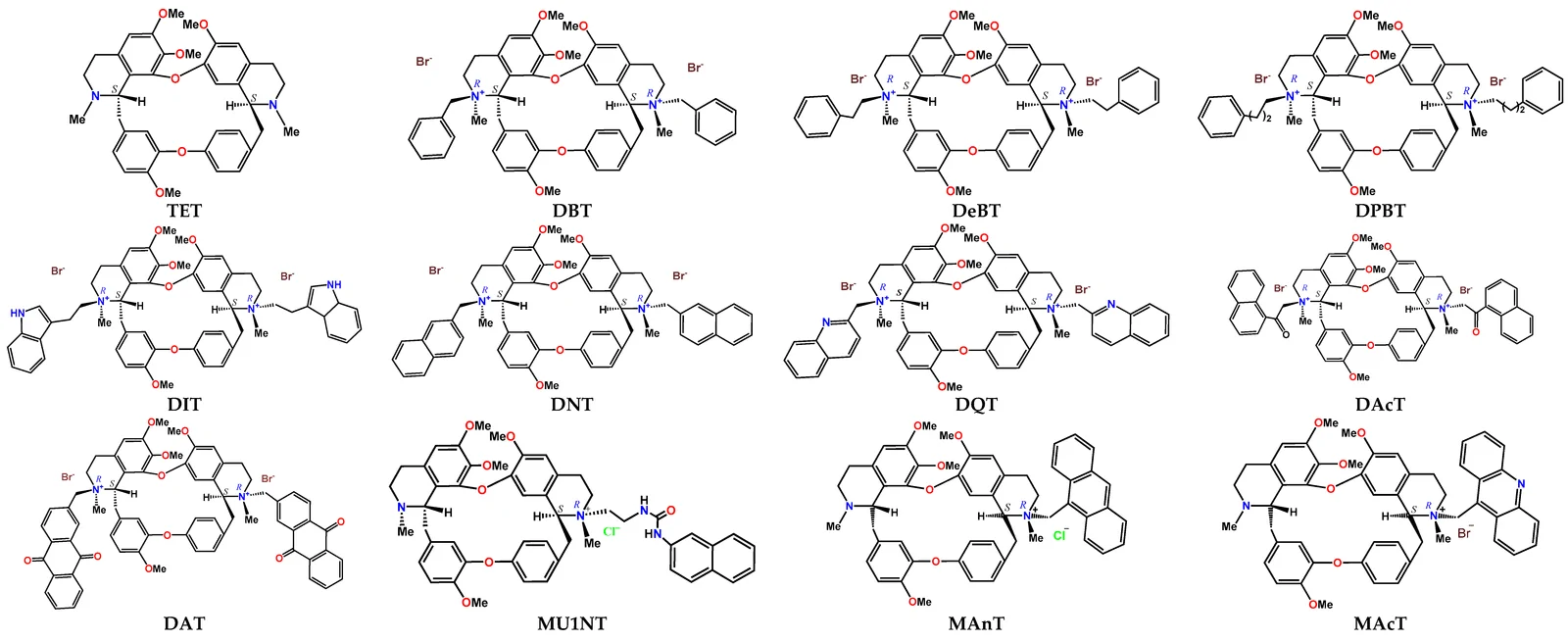
Chemical structures of tetrandrine and the di- and monosubstituted tetrandrine derivatives studied in this work. The chirality of the stereogenic centers in the isolated stereoisomers is indicated as *S* and *R*. Oxygen, nitrogen, bromine, and chlorine atoms are shown in red, blue, dark brown, and green, respectively.

**Figure 2:**
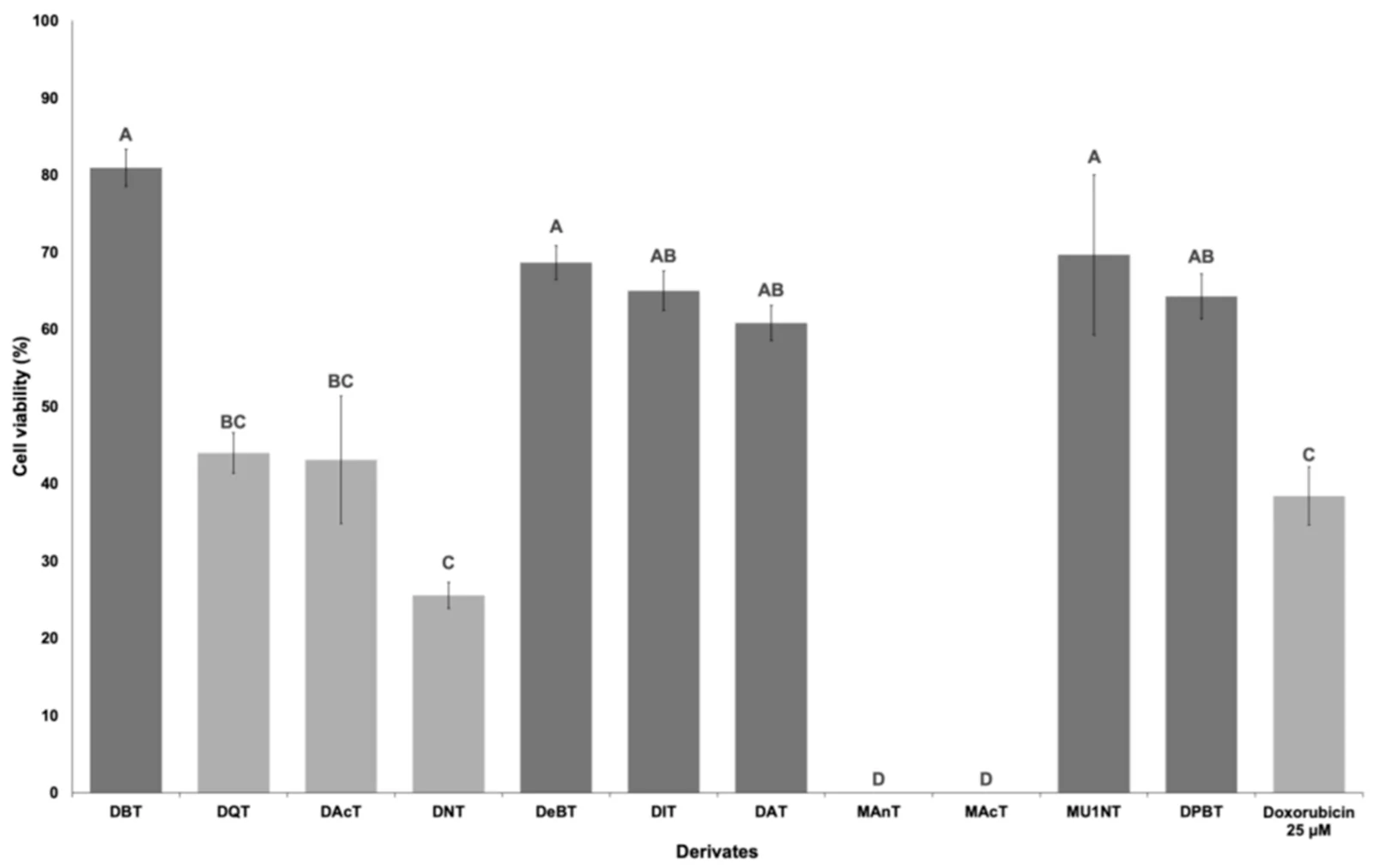
Cell viability percentages of non-cancerous human retinal pigment epithelial ARPE-19 cells treated with N-substituted tetrandrine derivatives (25 μM) for 48 h. Cell viability was determined using the MTT assay. Doxorubicin (25 μM) was used as a positive control. Data are presented as the mean ± standard error of three independent experiments performed in triplicate. Bars sharing at least one letter are not significantly different, whereas bars with no letters in common are significantly different according to the Tukey–Kramer test (*p* < 0.05).

**Figure 3:**
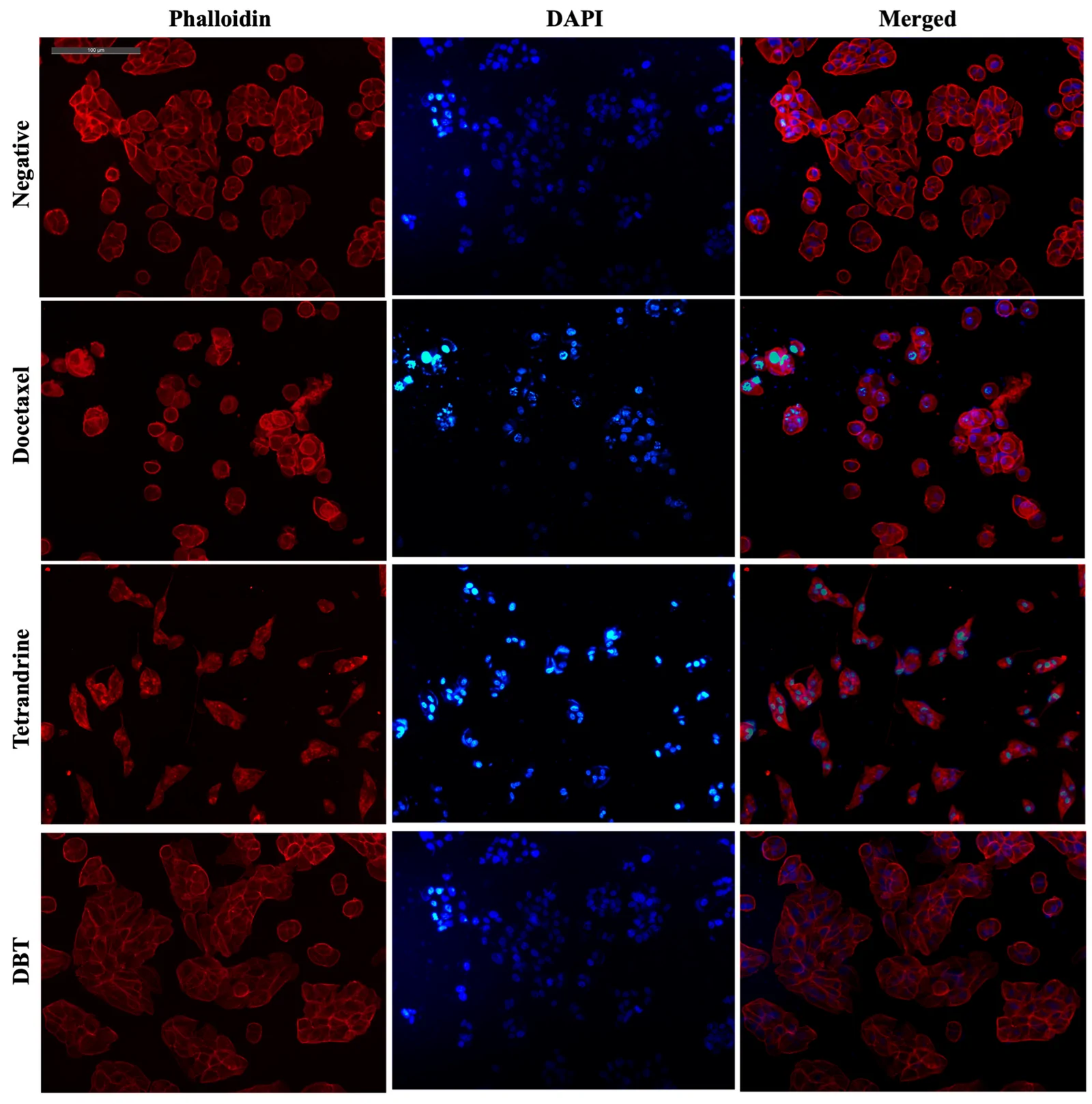
Fluorescence-microscopy analysis of morphological changes in non-cancerous ARPE-19 cells induced by **TET**, **DBT**, and docetaxel. Cells were treated for 24 h with **TET** (6.00 μM), **DBT** (9.89 μM), or docetaxel (84.31 μM), corresponding to their respective IC_50_ concentrations determined in 22Rv1 cells. Nuclear morphology was visualized by DAPI staining, and F-actin organization was examined using phalloidin staining. Representative images show the comparatively preserved cellular and nuclear morphology of ARPE-19 cells treated with **DBT**, relative to the alterations observed after treatment with **TET** and docetaxel. Cells were observed at 20×.

**Figure 4:**
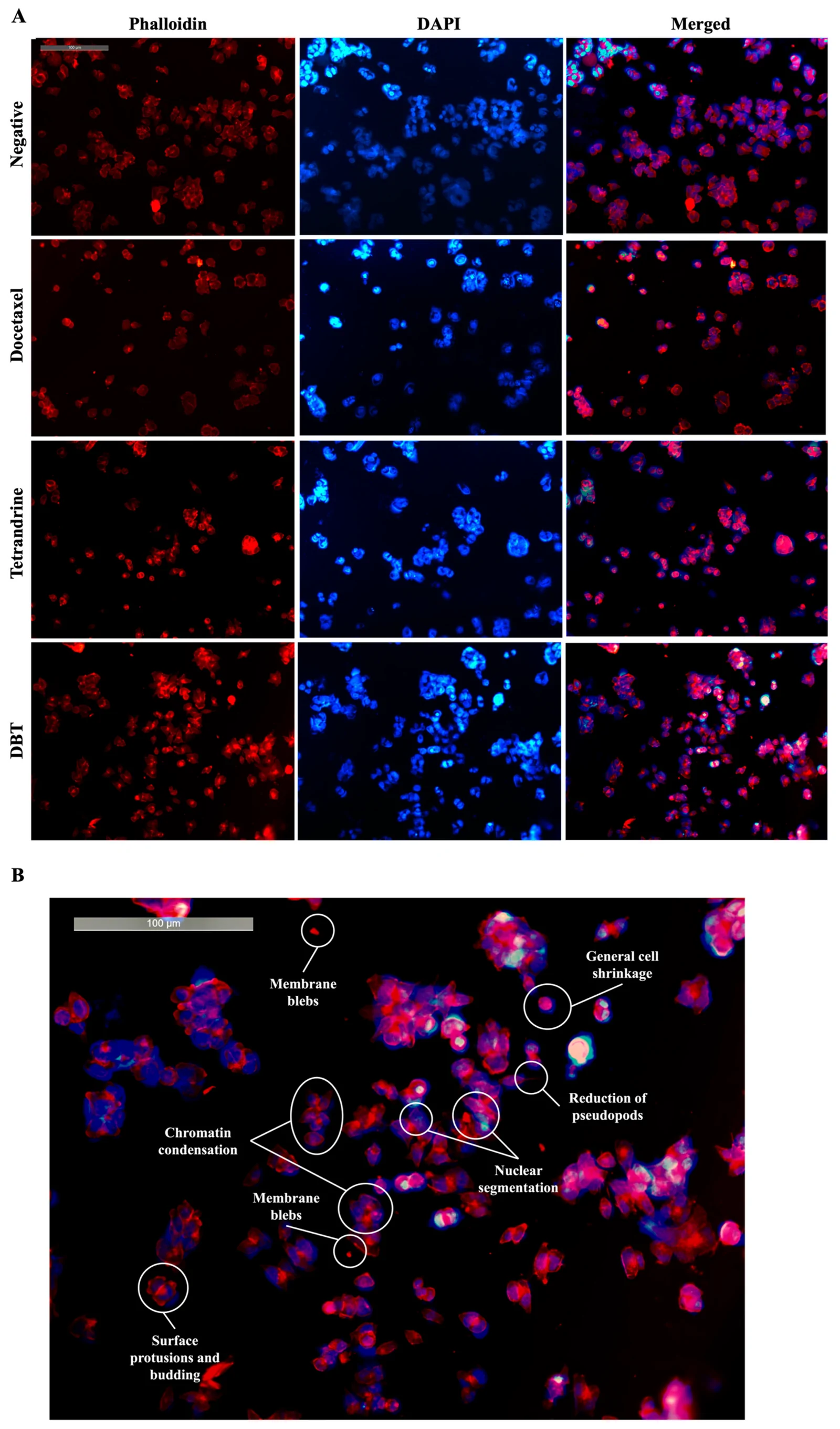
Fluorescence-microscopy analysis of morphological changes in 22Rv1 prostate carcinoma cells induced by **TET**, **DBT**, and docetaxel. Cells were treated for 24 h with **TET** (6.00 μM), **DBT** (9.89 μM), or docetaxel (84.31 μM), corresponding to their respective IC_50_ concentrations determined in 22Rv1 cells. Nuclear morphology was visualized by DAPI staining, and F-actin organization was examined using phalloidin staining. (**A**) Representative images of untreated and treated 22Rv1 cells. (**B**) Detailed morphological features observed in **DBT**-treated cells, including cell shrinkage, reduced pseudopodial extensions, membrane blebbing, chromatin condensation, nuclear fragmentation, and reorganization of the actin cytoskeleton. Cells were observed at 20×.

**Figure 5:**
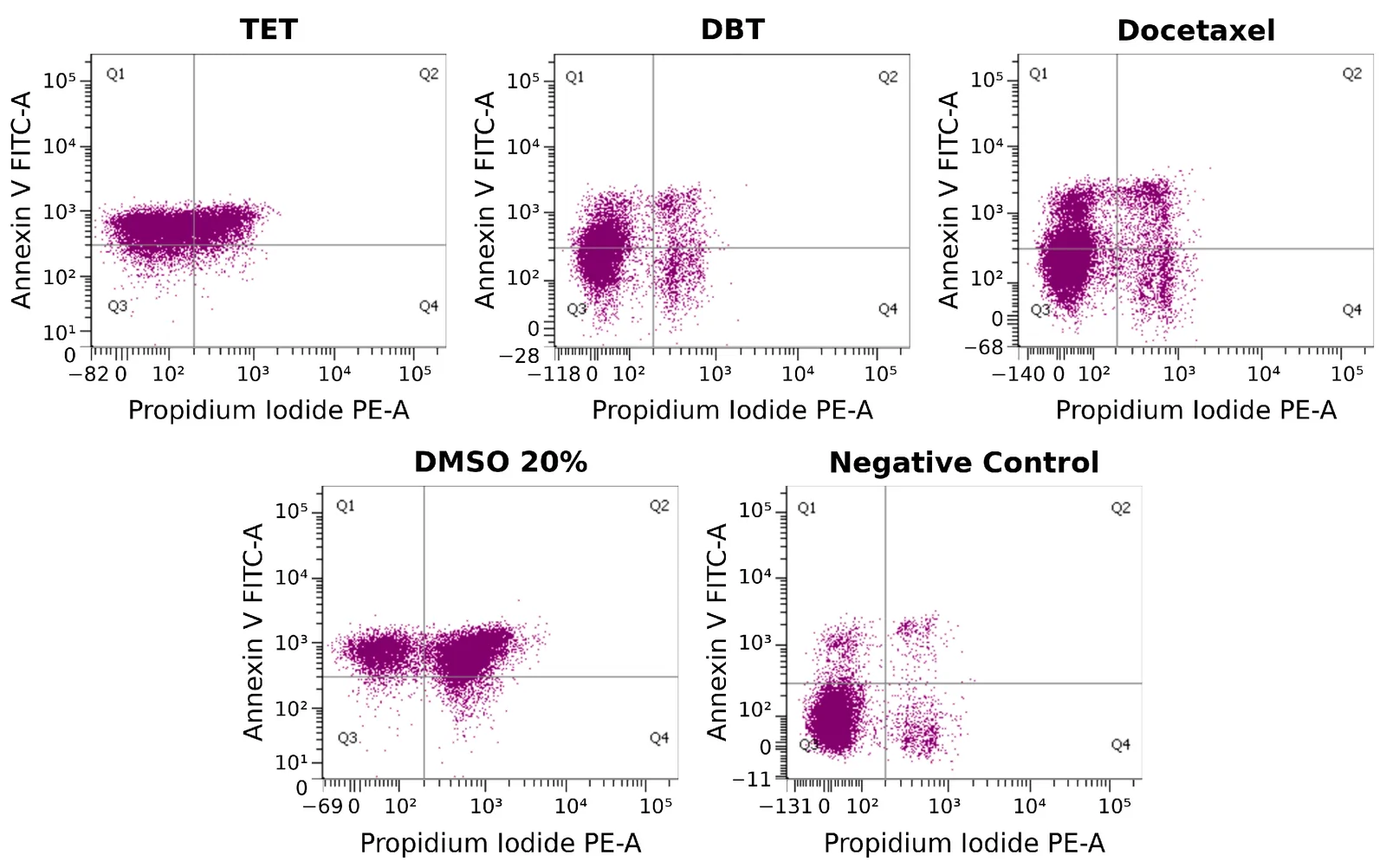
Representative flow cytometric analysis of cell death populations in 22Rv1 prostate cancer cells following 24 h of treatment at the respective 48 h MTT-derived IC_50_ concentrations of **TET** (6.00 μM), **DBT** (9.89 μM), and docetaxel (84.31 μM). Cells were stained with Annexin V-FITC and propidium iodide (PI). Quadrants represent early apoptotic cells (Q1, Annexin V^+^/PI^−^), late apoptotic cells (Q2, Annexin V^+^/PI^+^), viable cells (Q3, Annexin V^−^/PI^−^), and cells displaying a phenotype compatible with primary necrosis (Q4, Annexin V^−^/PI^+^). Untreated cells were included as the negative control, with 85.4% remaining viable, whereas cells exposed to 20% DMSO for 24 h were included as a positive cell death control.

**Figure 6:**
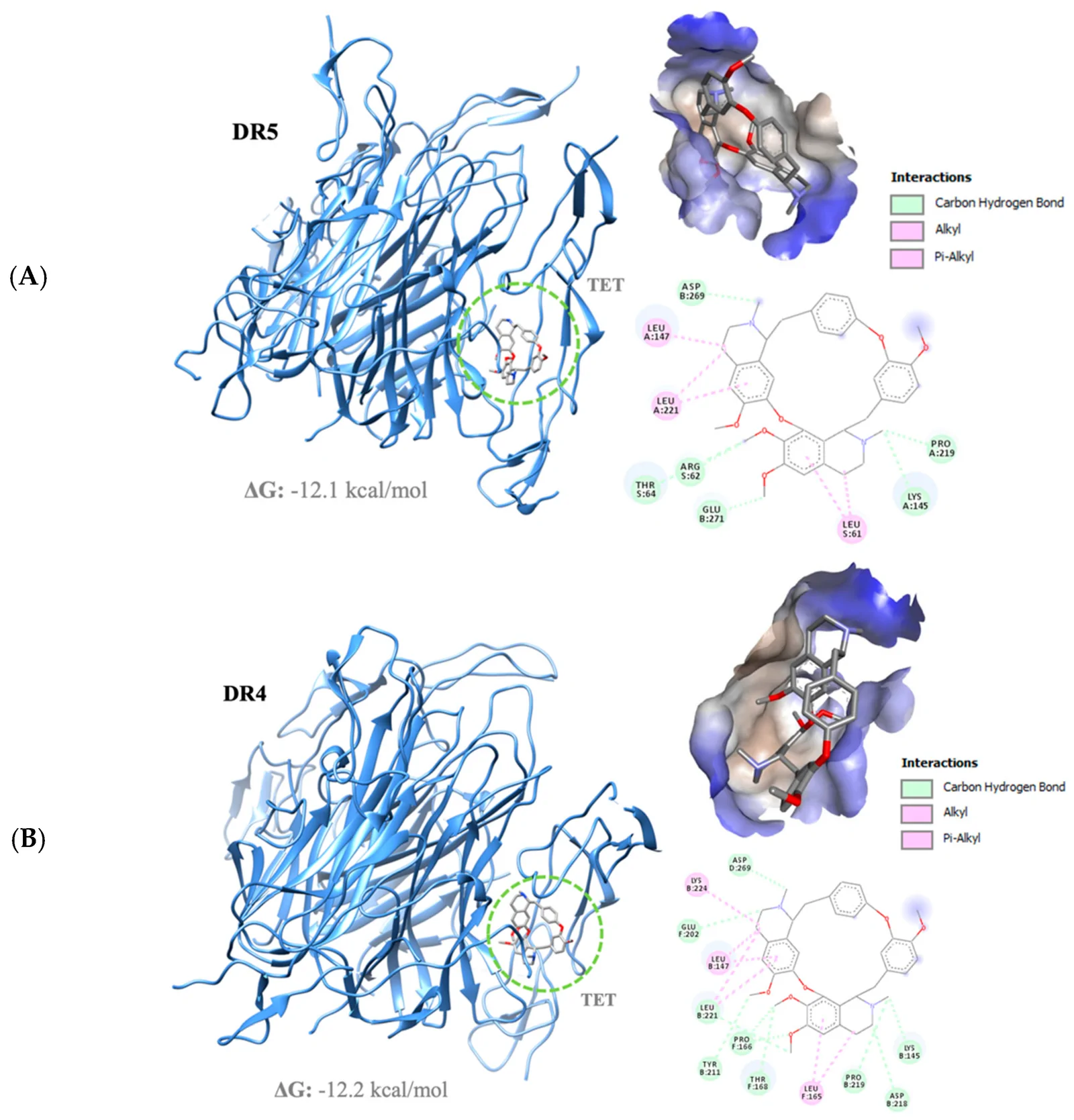

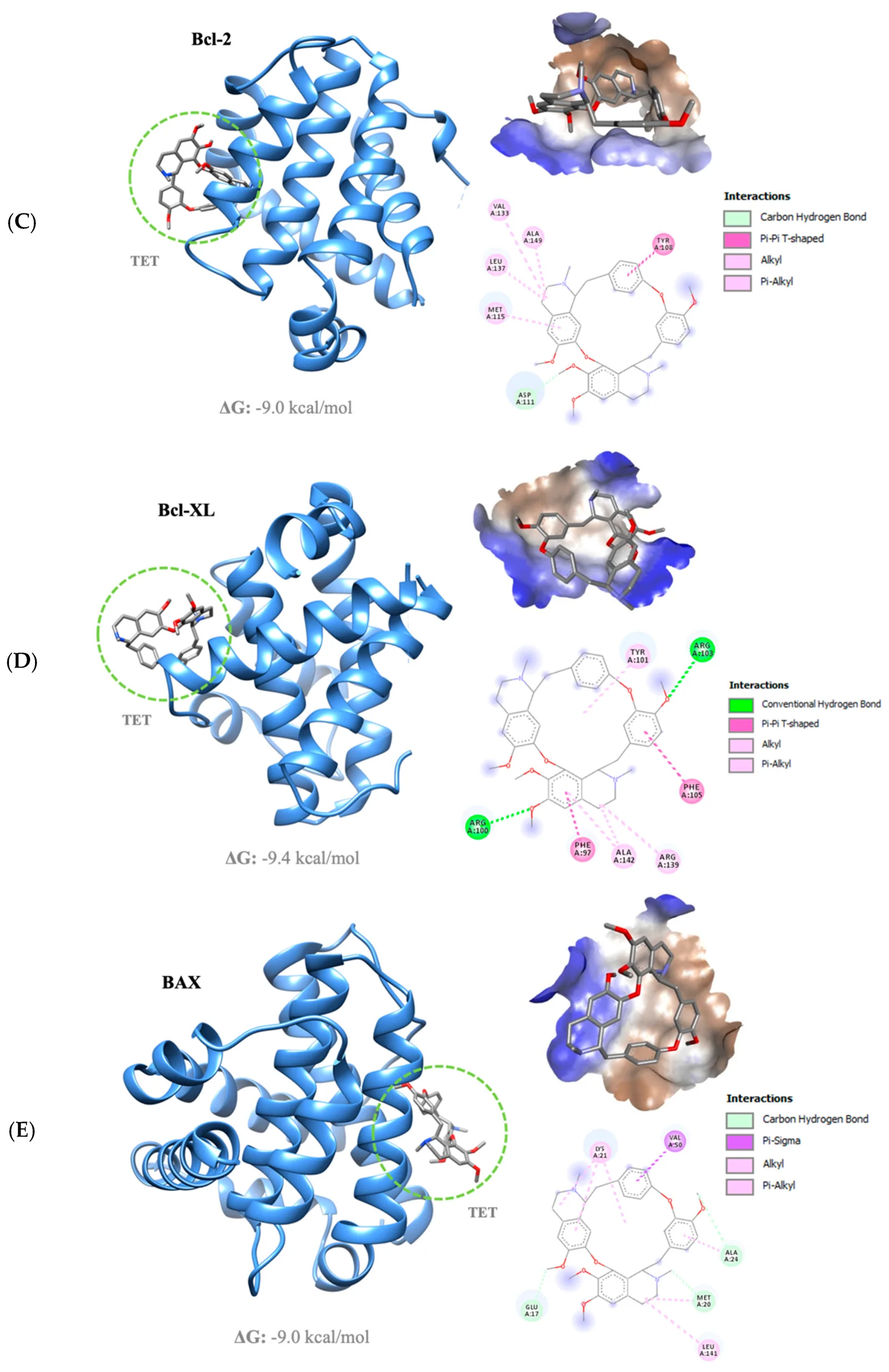
Top-ranked molecular docking poses of **TET** with apoptosis-related proteins: (**A**) DR5, (**B**) DR4, (**C**) Bcl-2, (**D**) Bcl-XL, and (**E**) BAX. For each complex, the predicted three-dimensional binding pose and corresponding two-dimensional interaction map are shown. The predicted binding energies obtained with AutoDock Vina were −12.10, −12.20, −9.00, −9.40, and −9.00 kcal/mol for DR5, DR4, Bcl-2, Bcl-XL, and BAX, respectively. Protein–ligand interactions were visualized using BIOVIA Discovery Studio Visualizer, version 2023. Oxygen and nitrogen atoms are shown in red and blue, respectively. Dashed interaction lines are color-coded according to the legend in each panel.

**Figure 7:**
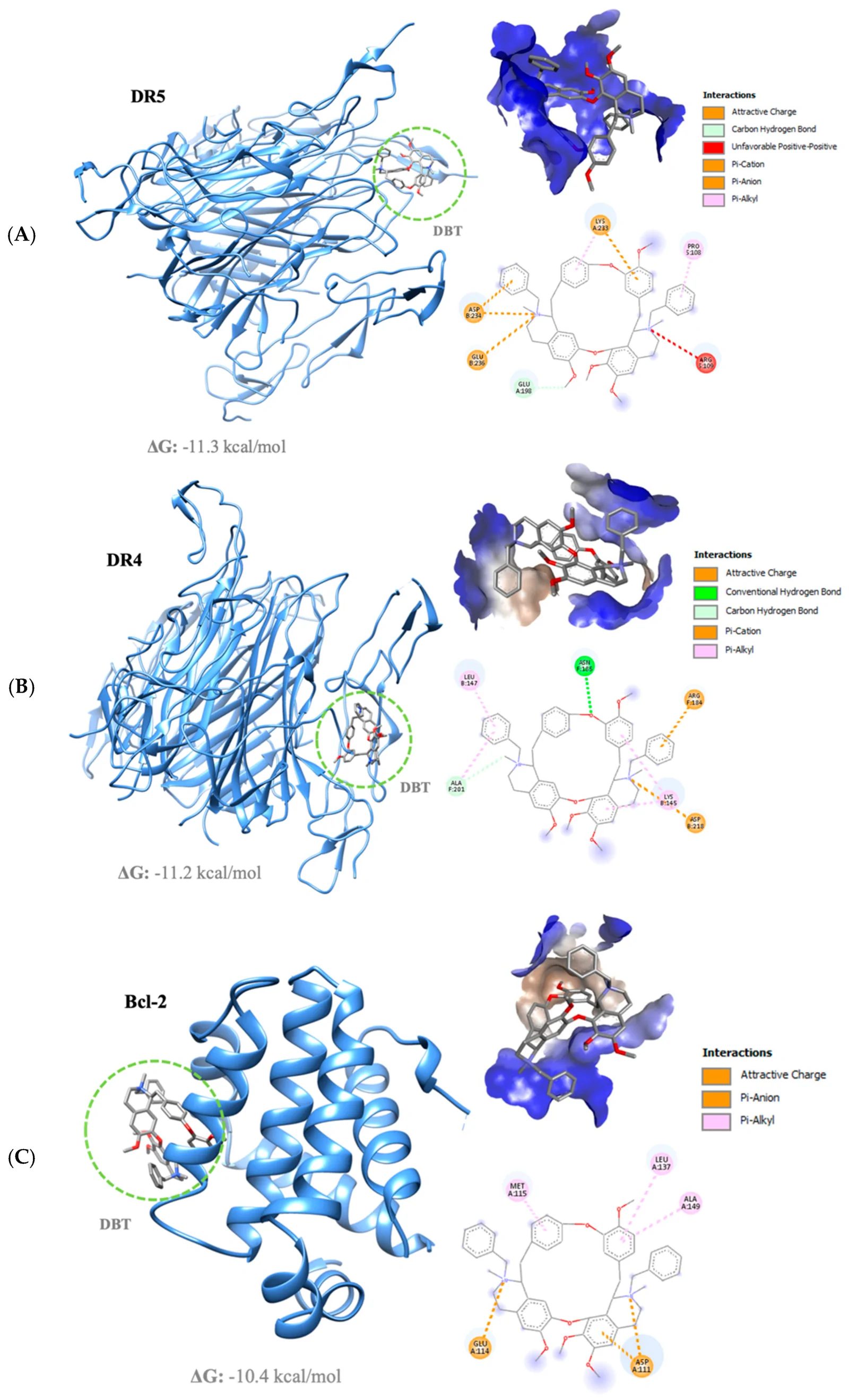

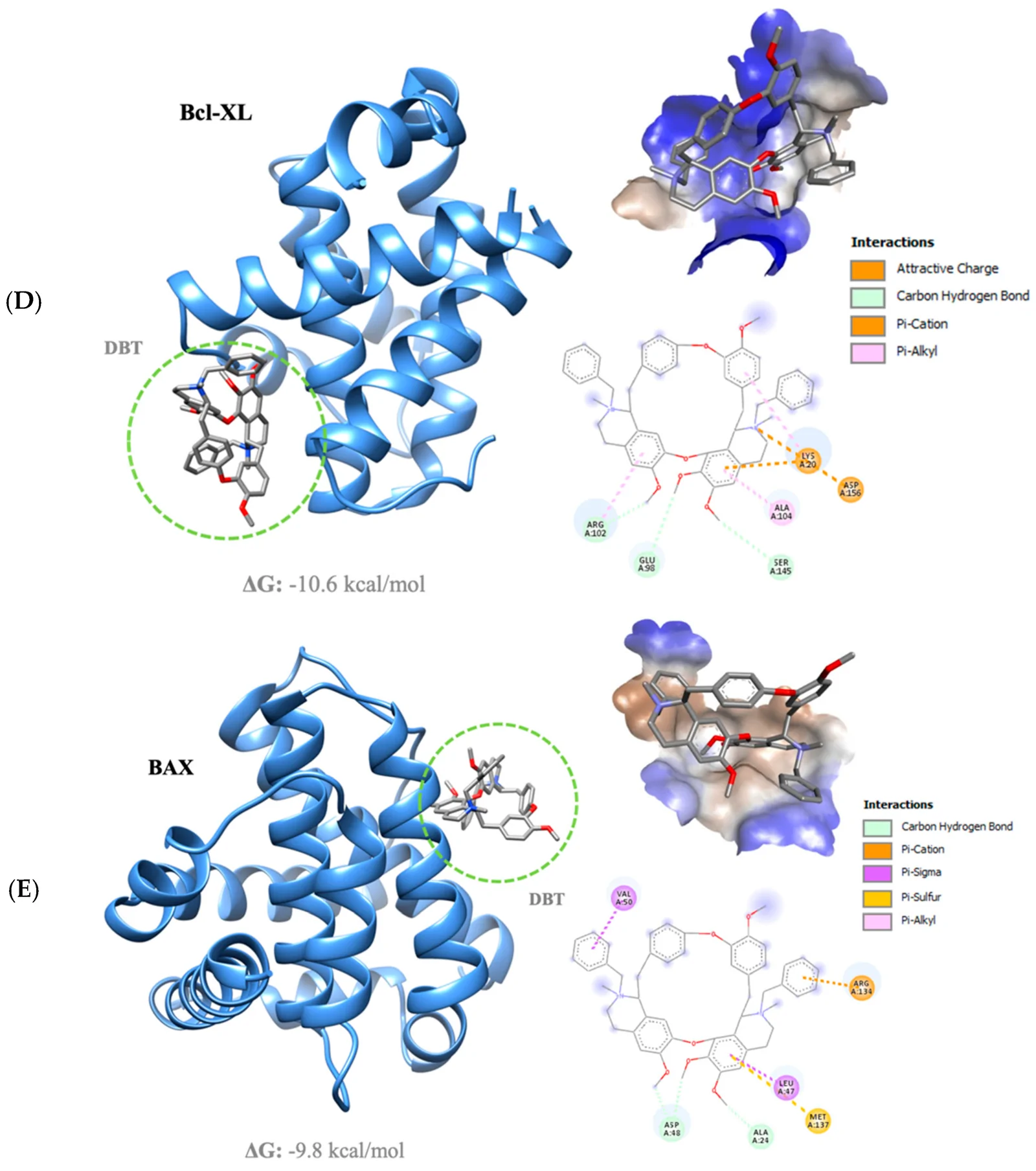
Top-ranked molecular docking poses of **DBT** with apoptosis-related proteins: (**A**) DR5, (**B**) DR4, (**C**) Bcl-2, (**D**) Bcl-XL, and (**E**) BAX. For each complex, the predicted three-dimensional binding pose and corresponding two-dimensional interaction map are shown. The predicted binding energies obtained with AutoDock Vina were −11.30, −11.20, −10.40, −10.60, and −9.80 kcal/mol for DR5, DR4, Bcl-2, Bcl-XL, and BAX, respectively. Protein–ligand interactions were visualized using BIOVIA Discovery Studio Visualizer, version 2023. Oxygen and nitrogen atoms are shown in red and blue, respectively. Dashed interaction lines are color-coded according to the legend in each panel.

**Table 1:** IC_50_ values of **TET** in non-cancerous ARPE-19 cells and five human cancer cell lines after 48 h of exposure.

Cell Line	Cell Type	IC_50_ of TET (μM)
ARPE-19	Non-cancerous retinal pigment epithelium	6.81 ± 1.79 ^cd^
22Rv1	Prostate carcinoma	6.00 ± 1.96 ^c^
A549	Lung adenocarcinoma	4.23 ± 1.62 ^d^
MDA-MB-231	Triple-negative breast cancer	15.99 ± 1.57 ^bc^
HCT-116	Colorectal carcinoma	15.88 ± 3.70 ^bc^
HeLa	Cervical cancer	17.09 ± 4.57 ^b^

Data are presented as the mean ± standard error of three independent experiments performed in triplicate. Different superscript letters within columns indicate significant differences (*p* < 0.05) by Tukey–Kramer’s test.

**Table 2:** Percentage values of cell viability for non-cancerous ARPE-19 cells and five human cancer cell lines after treatment with selected N-substituted tetrandrine derivatives for 48 h. Doxorubicin was used as a positive control.

Compound	Cell Viability (%) ± SEM					
	ARPE-19 [25 μM]	22Rv1 [95 μM]	A549 [25 μM]	MDA-MB-231 [15 μM]	HCT-116 [15 μM]	HeLa [17 μM]
**DBT**	80.90 ± 2.84 ^a^	5.59 ± 1.96 ^d^	70.85 ± 3.10 ^a^	100.00 ± 0.11 ^a^	69.11 ± 2.34 ^bc^	84.48 ± 4.08 ^a^
**DeBT**	68.61 ± 2.17 ^bc^	65.68 ± 1.12 ^a^	78.02 ± 0.72 ^a^	100.00 ± 2.00 ^abc^	78.55 ± 0.75 ^ab^	85.63 ± 0.36 ^ab^
**DIT**	64.97 ± 2.54 ^c^	18.77 ± 2.16 ^c^	56.43 ± 5.86 ^a^	100.00 ± 1.89 ^a^	62.09 ± 2.22 ^c^	63.11 ± 4.52 ^b^
**DAT**	60.78 ± 2.28 ^c^	25.01 ± 1.88 ^c^	63.31 ± 1.72 ^a^	87.24 ± 5.39 ^bc^	72.10 ± 3.03 ^abc^	74.66 ± 6.42 ^ab^
**MU1NT**	80.00 ± 0.21 ^ab^	4.72 ± 0.58 ^d^	13.34 ± 1.85 ^b^	84.78 ± 2.32 ^c^	76.57 ± 1.33 ^ab^	89.37 ± 5.54 ^a^
**DPBT**	64.23 ± 2.92 ^c^	45.03 ± 2.27 ^b^	80.35 ± 2.85 ^a^	100.00 ± 0.91 ^ab^	83.47 ± 0.94 ^a^	93.70 ± 6.54 ^ab^
Doxorubicin	38.38 ± 3.75 ^d^	7.24 ± 0.21 ^d^	16.12 ± 7.52 ^b^	3.28 ± 1.00 ^d^	0.00 ± 1.46 ^d^	0.00 ± 0.44 ^c^

Values represent cell viability percentages and are expressed as the mean ± standard error of three independent experiments, each performed in triplicate. Statistical comparisons were performed separately for each cell line using the Tukey–Kramer test (*p* < 0.05). Different superscript letters within each column indicate significant differences.

**Table 3:** IC_50_ values and selectivity indices of **TET**, **DBT**, **MU1NT**, and docetaxel in non-cancerous ARPE-19 cells and selected human cancer cell lines after 48 h of exposure.

Cell Line	Compound	IC_50_ (μM)	Selectivity Index
ARPE-19	**TET**	6.81 ± 1.79 ^d^	N/A
	**DBT**	45.44 ± 1.37 ^c^	N/A
	**MU1NT**	71.89 ± 3.33 ^b^	N/A
	Docetaxel	>200 ^a^	N/A
22Rv1	**TET**	6.00 ± 1.96 ^c^	1.13
	**DBT**	9.89 ± 1.08 ^c^	4.59
	**MU1NT**	28.00 ± 3.85 ^b^	2.57
	Docetaxel	84.31 ± 4.48 ^a^	>2.37
A549	**TET**	4.23 ± 1.62 ^c^	1.61
	**MU1NT**	65.73 ± 4.46 ^a^	1.09

IC_50_ values are expressed as the mean ± standard error of three independent experiments, each performed in triplicate. The selectivity index (*SI*) was calculated as the IC_50_ in non-cancerous ARPE-19 cells divided by the IC_50_ in cancer cells; therefore, *SI* values are not applicable to the ARPE-19 reference cell line (indicated as N/A). Different superscript letters within each cell line indicate significant differences according to the Tukey–Kramer test (*p* < 0.05). The IC_50_ of docetaxel in ARPE-19 cells was not reached at the highest concentration evaluated; therefore, its *SI* in 22Rv1 cells is reported as a lower bound. N/A, not applicable.

**Table 4:** Flow cytometric analysis of cell death populations in 22Rv1 prostate cancer cells following 24 h of treatment with **TET**, **DBT**, or docetaxel at concentrations corresponding to their respective 48 h IC_50_ values determined by the MTT assay.

Treatment	Viable Cells (%)	Early Apoptosis (%)	Late Apoptosis (%)	Total Apoptosis (%)	Primary Necrosis (%)
**TET**	10.90 ± 1.27	59.70 ± 2.69	26.60 ± 3.96	86.30 ± 1.27	2.85 ± 0.07
**DBT**	59.20 ± 6.60	29.10 ± 3.80	5.67 ± 1.67	34.77 ± 5.13	6.07 ± 2.03
Docetaxel	62.07 ± 4.48	25.37 ± 2.47	6.80 ± 1.39	32.17 ± 3.07	5.87 ± 1.42

Cell populations were classified by Annexin V-FITC/PI staining as viable (Annexin V^−^/PI^−^), early apoptotic (Annexin V^+^/PI^−^), late apoptotic (Annexin V^+^/PI^+^), or compatible with primary necrosis (Annexin V^−^/PI^+^). Total apoptosis was calculated separately for each technical replicate as the sum of the early- and late apoptotic populations. Values are expressed as the mean ± standard deviation (SD) of the available technical replicates from a single experiment (**TET**, *n* = 2; **DBT** and docetaxel, *n* = 3). The data were analyzed descriptively; no inferential statistical comparisons were performed.

**Table 5:** Predicted binding energies (Δ*G*, kcal/mol) for the top-ranked molecular docking poses of **TET** and **DBT** with selected apoptosis-related proteins.

Protein Target	Compound	Binding Energy (kcal/mol)
DR5	**TET**	−12.10
	**DBT**	−11.30
DR4	**TET**	−12.20
	**DBT**	−11.20
Bcl-2	**TET**	−9.00
	**DBT**	−10.40
Bcl-XL	**TET**	−9.40
	**DBT**	−10.60
BAX	**TET**	−9.00
	**DBT**	−9.80

**Table 6:** Site-specific docking scores and redocking validation for Bcl-2.

Protein/PDB	Ligand	Role	Score (kcal/mol)	RMSD (Å)
Bcl-2/6O0K	Venetoclax	Co-crystallized reference	−12.2	0.55
Bcl-2/6O0K	**TET**	Test ligand	−7.8	N/A
Bcl-2/6O0K	**DBT**	Test ligand	−8.3	N/A

The targeted Bcl-2 scores are not directly comparable with the blind docking values reported in Table 5. RMSD was calculated using symmetry-corrected ligand heavy atoms in the receptor coordinate frame without post-docking fitting. N/A, not applicable.

## Data Availability

The data presented in this article are included in the article and its Supplementary Information. Public cell line RNA expression data used in the additional analysis are available from the Human Protein Atlas, version 25.1, and the NCBI Gene Expression Omnibus under accession number GSE159235. The protein structures used for molecular docking are available from the Protein Data Bank under accession codes 1D0G (DR5), 5CIR (DR4), 6O0K (Bcl-2), 1R2D (Bcl-XL), and 6EB6 (BAX).

## Supplementary Materials

The following supporting information can be downloaded at https://www.mdpi.com/article/10.3390/molecules31193572/s1: Figures S1–S4: 1H and 13C NMR, ESI(+) mass, and FTIR-ATR spectra of MU1NT; Figures S5–S7: 1H and 13C NMR and ESI(+) mass spectra of DPBT; Figures S8–S11: 1H and 13C NMR, ESI(+) mass, and FTIR-ATR spectra of U1N; Figure S12: Representative bright-field microscopy images of ARPE-19 cells after treatment with selected compounds; Figures S13 and S14: Representative concentration–response profiles of TET and DBT, respectively, in 22Rv1 cells; Supplementary Method for Figure S15: Generation of the redocking validation plot; Figure S15: Validation of the targeted Bcl-2 docking protocol by redocking of venetoclax; Tables S1 and S2: Baseline RNA expression of apoptosis-related docking targets in the cancer cell lines and ARPE-19 controls, respectively.

## References

[1] World Health Organization (WHO) Cancer. https://www.who.int/news-room/fact-sheets/detail/cancer.

[2] Longley D.B., Johnston P.G. (2005). Molecular mechanisms of drug resistance. J. Pathol..

[3] Luan F., He X., Zeng N. (2020). Tetrandrine: A review of its anticancer potentials, clinical settings, pharmacokinetics and drug delivery systems. J. Pharm. Pharmacol..

[4] Hui Z., Wen H., Zhu J., Deng H., Jiang X., Ye X.-Y., Wang L., Xie T., Bai R. (2024). Discovery of Plant-Derived Anti-Tumor Natural Products: Potential Leads for Anti-Tumor Drug Discovery. Bioorg. Chem..

[5] Ghosh S., Das S.K., Sinha K., Ghosh B., Sen K., Ghosh N., Sil P.C. (2024). The Emerging Role of Natural Products in Cancer Treatment. Arch. Toxicol..

[6] Mishra P.S., Kaushik N., Mishra R., Koul S., Sagar S. (2024). Natural Alkaloids and Mechanisms for Anti-Cancer Action: A Review. Curr. Bioact. Compd..

[7] Bhagya N., Chandrashekar K.R. (2018). Tetrandrine and cancer—An overview on the molecular approach. Biomed. Pharmacother..

[8] Gong L., Liu H., Xu B., Yu T., Wang Y., Niu S.-L., Zeng R., Ouyang Q. (2024). Insights on exploring the therapeutic potential and structural modification of tetrandrine. Future Med. Chem..

[9] Yoo S.-M., Oh S.-H., Lee S.-J., Lee B.-W., Ko W.-G., Moon C.-K., Lee B.-H. (2002). Inhibition of proliferation and induction of apoptosis by tetrandrine in HepG2 cells. J. Ethnopharmacol..

[10] Qiu W., Su M., Xie F., Ai J., Ren Y., Zhang J., Guan R., He W., Gong Y., Guo Y. (2014). Tetrandrine blocks autophagic flux and induces apoptosis via energetic impairment in cancer cells. Cell Death Dis..

[11] Liu W., Kou B., Ma Z.-K., Tang X.-S., Lv C., Ye M., Chen J.-Q., Li L., Wang X.-Y., He D.-L. (2015). Tetrandrine suppresses proliferation, induces apoptosis, and inhibits migration and invasion in human prostate cancer cells. Asian J. Androl..

[12] Lin Y.-J., Peng S.-F., Lin M.-L., Kuo C.-L., Lu K.-W., Liao C.-L., Ma Y.-S., Chueh F.-S., Liu K.-C., Yu F.-S. (2016). Tetrandrine induces apoptosis of human nasopharyngeal carcinoma NPC-TW 076 cells through reactive oxygen species accompanied by an endoplasmic reticulum stress signaling pathway. Molecules.

[13] Yu F.-S., Yu C.-S., Chen J.-C., Yang J.-L., Lu H.-F., Chang S.-J., Lin M.-W., Chung J.-G. (2016). Tetrandrine induces apoptosis via caspase-8, -9, and -3 and poly(ADP-ribose) polymerase-dependent pathways and autophagy through Beclin-1/LC3-I/II signaling pathways in human oral cancer HSC-3 cells. Environ. Toxicol..

[14] Shishodia G., Koul S., Dong Q., Koul H.K. (2018). Tetrandrine induces death receptors Apo Trail R1 (DR4) and Apo Trail R2 (DR5) and sensitizes prostate cancer cells to TRAIL-induced apoptosis. Mol. Cancer Ther..

[15] Tan Y., Zhu Q., Yang M., Yang F., Zeng Q., Jiang Z., Li D. (2024). Tetrandrine activates STING/TBK1/IRF3 pathway to potentiate anti-PD-1 immunotherapy efficacy in non-small-cell lung cancer. Pharmacol. Res..

[16] Xin G., Hou Y., Liu Y., Li H. (2025). Tetrandrine and adriamycin reverse multidrug resistance by regulating NLRP3/Caspase-1/GSDMD signaling in human breast cancer cells. Eur. J. Pharmacol..

[17] Mo L., Zhang F., Chen F., Xia L., Huang Y., Mo Y., Zhang L., Huang D., He S., Deng J. (2022). Progress on structural modification of tetrandrine with a wide range of pharmacological activities. Front. Pharmacol..

[18] Hu S.-C., Yang J., Chen C., Song J.-R., Pan W.-D. (2020). Design, Synthesis of Novel Tetrandrine-14-L-Amino Acid and Tetrandrine-14-L-Amino Acid-Urea Derivatives as Potential Anti-Cancer Agents. Molecules.

[19] Wang Y.-C., Zhang R.-H., Hu S.-C., Zhang H., Yang D., Zhang W.-L., Zhao Y.-L., Cui D.-B., Li Y.-J., Pan W.-D. (2022). Design, Synthesis, and Biological Evaluation of N14-Amino Acid-Substituted Tetrandrine Derivatives as Potential Antitumor Agents against Human Colorectal Cancer. Molecules.

[20] Jiang T., Xie G., Zeng Z., Lan J., Liu H., Li J., Ren H., Chen T., Pan W. (2023). A Novel Class of C14-Sulfonate-Tetrandrine Derivatives as Potential Chemotherapeutic Agents for Hepatocellular Carcinoma. Front. Chem..

[21] Ling J., Li X., Wang M., Zhang C., Liu Y., Zhang X., Liu C., Ren Q., Zeng Y., Wang C. (2024). Novel Sulfonyl-Substituted Tetrandrine Derivatives for Colon Cancer Treatment by Inducing Mitochondrial Apoptosis and Inhibiting PI3K/AKT/mTOR Pathway. Bioorg. Chem..

[22] Zeng R., Yang X.-M., Li H.-W., Li X., Guan Y., Yu T., Yan P., Yuan W., Niu S.-L., Gu J. (2023). Simplified Derivatives of Tetrandrine as Potent and Specific P-gp Inhibitors to Reverse Multidrug Resistance in Cancer Chemotherapy. J. Med. Chem..

[23] Jiang T., Shi L., Peng X., Zheng S., Chen Q., Lan J., Pan W. (2025). Investigating C7 Modified Tetrandrine Derivatives for Synthesis, Anti-Hepatocellular Carcinoma Activity, and Mechanistic Insights. Sci. Rep..

[24] Lara K.O., Godoy-Alcántar C., Rivera I.L., Eliseev A.V., Yatsimirsky A.K. (2001). Complexation of Dicarboxylates and Phosphates by a Semisynthetic Alkaloid-Based Cyclophane in Water. J. Phys. Org. Chem..

[25] Lara K.O., Godoy-Alcántar C., Eliseev A.V., Yatsimirsky A.K. (2004). Recognition of α-Amino Acid Derivatives by N,N′-Dibenzylated S,S-(+)-Tetrandrine. Org. Biomol. Chem..

[26] Lara K.O., Godoy-Alcántar C., Eliseev A.V., Yatsimirsky A.K. (2005). Ester Cleavage by S,S-(+)-Tetrandrine Bearing Attached Thiol Groups. ARKIVOC.

[27] Moreno-Corral R., Lara K.O. (2008). Complexation Studies of Nucleotides by Tetrandrine Derivatives Bearing Anthraquinone and Acridine Groups. Supramol. Chem..

[28] Wong-Molina A., Lara K., Sánchez M., Burboa M.G., Gutiérrez-Millán L.E., Marín J.L., Valdez M.A. (2008). Interaction of Calf Thymus DNA with a Cationic Tetrandrine Derivative at the Air–Water Interface. J. Biomed. Nanotechnol..

[29] Calvillo-Páez V., Sotelo-Mundo R.R., Leyva-Peralta M., Gálvez-Ruiz J.C., Corona-Martínez D., Moreno-Corral R., Escobar-Picos R., Höpfl H., Juárez-Sánchez O., Lara K.O. (2018). Synthesis, Spectroscopic, Physicochemical and Structural Characterization of Tetrandrine-Based Macrocycles Functionalized with Acridine and Anthracene Groups: DNA Binding and Anti-Proliferative Activity. Chem. Biol. Interact..

[30] Escobar-Picos R., Vasquez-Ríos M.G., Sotelo-Mundo R.R., Jancik V., Martínez-Otero D., Calvillo-Páez V., Höpfl H., Ochoa Lara K. (2019). A Chiral Bis-Naphthylated Tetrandrine Dibromide: Synthesis, Self-Assembly into an Organic Framework Based on Nanosized Spherical Cages, and Inclusion Studies. ChemPlusChem.

[31] González-Martínez S.M., Valencia-Ochoa D.P., Gálvez-Ruiz J.C., Leyva-Peralta M.A., Juárez-Sánchez O., Islas-Osuna M.A., Calvillo-Páez V.I., Höpfl H., Íñiguez-Palomares R., Rocha-Alonzo F. (2022). DNA-Binding Properties of Bis-N-Substituted Tetrandrine Derivatives. ACS Omega.

[32] Calvillo-Páez V.I., Plascencia-Jatomea M., Ochoa-Terán A., Del-Toro-Sánchez C.L., González-Vega R.I., González-Martínez S.M., Ochoa Lara K. (2023). Tetrandrine Derivatives as Promising Antibacterial Agents. ACS Omega.

[33] Calvillo-Páez V.I., Ochoa-Terán A., Gálvez-Ruiz J.C., Hernández-Zazueta M.S., Del-Toro-Sánchez C.L., Rascón-Valenzuela L.A., Juárez-Sánchez J.O., Gomez-Vega P.J., Plascencia-Jatomea M., Ochoa Lara K. (2026). N,N′-Disubstituted Tetrandrine Derivatives: Enhanced Antibacterial Activity via Double Quaternization. ACS Omega.

[34] Tao L.-J., Zhou X.-D., Shen C.-C., Liang C.-Z., Liu B., Tao Y., Tao H.-M. (2014). Tetrandrine induces apoptosis and triggers a caspase cascade in U2-OS and MG-63 cells through the intrinsic and extrinsic pathways. Mol. Med. Rep..

[35] Wang P., Zhang R., Liu M., Gao J., Liu X., Wang L., Wu Y., Han L., Deng Z., Chang T. (2025). Integrated network pharmacology, molecular docking and pharmacodynamic studies reveal protective effects and mechanisms of tetrandrine against sepsis-induced acute lung injury. Biochem. Biophys. Res. Commun..

[36] Ma R., Huang X., Sun D., Wang J., Xue C., Ye Q. (2024). Tetrandrine alleviates silica-induced pulmonary fibrosis through PI3K/AKT pathway: Network pharmacology investigation and experimental validation. Inflammation.

[37] Orencio S.S.M., Villaflores O.B., Saputra F., Audira G., Hsiao C.-D., Vasquez R.D. (2026). Dual potential of tetrandrine: Antiangiogenic efficacy and safety liabilities revealed through *In silico*, *in vitro*, and zebrafish models. Food Chem. Toxicol..

[38] Johnston T.P., McCaleb G.S., Opliger P.S., Montgomery J.A. (1966). The Synthesis of Potential Anticancer Agents. XXXVI. N-Nitrosoureas. II. Haloalkyl Derivatives. J. Med. Chem..

[39] Dissanayake I.H., Bandaranayake U., Keerthirathna L.R., Manawadu C., Silva R.M., Mohamed B., Ali R., Peiris D.C. (2022). Integration of In Vitro and In Silico Analysis of *Caulerpa racemosa* Against Antioxidant, Antidiabetic, and Anticancer Activities. Sci. Rep..

[40] Tannock I.F., de Wit R., Berry W.R., Horti J., Pluzanska A., Chi K.N., Oudard S., Théodore C., James N.D., Turesson I. (2004). Docetaxel plus Prednisone or Mitoxantrone plus Prednisone for Advanced Prostate Cancer. N. Engl. J. Med..

[41] Dyshlovoy S.A., Pelageev D.N., Jakob L.S., Borisova K.L., Hauschild J., Busenbender T., Kaune M., Khmelevskaya E.A., Graefen M., Bokemeyer C. (2021). Activity of New Synthetic (2-Chloroethylthio)-1,4-naphthoquinones in Prostate Cancer Cells. Pharmaceuticals.

[42] Dyshlovoy S.A., Shubina L.K., Makarieva T.N., Hauschild J., Strewinsky N., Guzii A.G., Menshov A.S., Popov R.S., Grebnev B.B., Busenbender T. (2022). New Diterpenes from the Marine Sponge *Spongionella* sp. Overcome Drug Resistance in Prostate Cancer by Inhibition of P-Glycoprotein. Sci. Rep..

[43] Dyshlovoy S.A., Busenbender T., Hauschild J., Girich E.V., Kriegs M., Hoffer K., Graefen M., Yurchenko A.N., Bokemeyer C., von Amsberg G. (2022). Cytotoxic N-Methylpretrichodermamide B Reveals Anticancer Activity and Inhibits P-Glycoprotein in Drug-Resistant Prostate Cancer Cells. Mar. Drugs.

[44] Kerr J.F.R., Wyllie A.H., Currie A.R. (1972). Apoptosis: A Basic Biological Phenomenon with Wide-Ranging Implications in Tissue Kinetics. Br. J. Cancer.

[45] Croft D.R., Coleman M.L., Li S., Robertson D., Sullivan T., Stewart C.L., Olson M.F. (2005). Actin–Myosin-Based Contraction Is Responsible for Apoptotic Nuclear Disintegration. J. Cell Biol..

[46] Elmore S. (2007). Apoptosis: A Review of Programmed Cell Death. Toxicol. Pathol..

[47] Trujillo-Ruiz H.E., Leal-Rodríguez D.G., Santacruz-Ortega H.d.C., Martínez-Cruz O., De La Reé-Rodríguez S.C., Burgos-Hernández A., Silva-Campa E., Carbonell-Barrachina Á.A., López-Saiz C.M. (2026). Chemical Characterization and Antiproliferative Evaluation of Compounds Isolated from White Shrimp (*Penaeus vannamei*) By-Products. Biophysica.

[48] Zhao Y., Cheng X., Wang G., Liao Y., Qing C. (2020). Linalool inhibits 22Rv1 prostate cancer cell proliferation and induces apoptosis. Oncol. Lett..

[49] Chaudhary P., Vishwanatha J.K. (2014). c-Jun NH_2_-terminal kinase-induced proteasomal degradation of c-FLIP_L/S_ and Bcl_2_ sensitize prostate cancer cells to Fas- and mitochondria-mediated apoptosis by tetrandrine. Biochem. Pharmacol..

[50] Pilling A., Kim S.-H., Hwang C. (2022). Androgen receptor negatively regulates mitotic checkpoint signaling to induce docetaxel resistance in castration-resistant prostate cancer. Prostate.

[51] Birkinshaw R.W., Gong J.-N., Luo C.S., Lio D., White C.A., Anderson M.A., Blombery P., Lessene G., Majewski I.J., Thijssen R. (2019). Structures of BCL-2 in complex with venetoclax reveal the molecular basis of resistance mutations. Nat. Commun..

[52] Human Protein Atlas Cell Line Data, Version 25.1, Ensembl Version 109. https://www.proteinatlas.org/humanproteome/cell%2Bline/data.

[53] NCBI Gene Expression Omnibus GSE159235: Differential Expression of Genes in HCMV-Infected or Zeocin-Treated ARPE-19 Cells. https://www.ncbi.nlm.nih.gov/geo/query/acc.cgi?acc=GSE159235.

[54] Nusinow D.P., Szpyt J., Ghandi M., Rose C.M., McDonald E.R., Kalocsay M., Jané-Valbuena J., Gelfand E., Schweppe D.K., Jedrychowski M. (2020). Quantitative proteomics of the Cancer Cell Line Encyclopedia. Cell.

[55] Gonçalves E., Poulos R.C., Cai Z., Barthorpe S., Manda S.S., Lucas N., Beck A., Bucio-Noble D., Dausmann M., Hall C. (2022). Pan-cancer proteomic map of 949 human cell lines. Cancer Cell.

[56] Mosmann T. (1983). Rapid colorimetric assay for cellular growth and survival: Application to proliferation and cytotoxicity assays. J. Immunol. Methods.

[57] Van Vuuren R.J., Botes M., Jurgens T., Joubert A.M., van den Bout I. (2019). Novel Sulphamoylated 2-Methoxy Estradiol Derivatives Inhibit Breast Cancer Migration by Disrupting Microtubule Turnover and Organization. Cancer Cell Int..

[58] Gilmore C.J., Bryan R.F., Kupchan S.M. (1976). Conformation and Reactivity of the Macrocyclic Tumor-Inhibitory Alkaloid Tetrandrine. J. Am. Chem. Soc..

[59] Zhou Z., Tong H.H.Y., Li L., Shek F.L.Y., Lv Y., Zheng Y. (2014). Synthesis, crystal structures and phase transformation of the new solid-state forms of tetrandrine. RSC Adv..

[60] Trott O., Olson A.J. (2010). AutoDock Vina: Improving the speed and accuracy of docking with a new scoring function, efficient optimization, and multithreading. J. Comput. Chem..

[61] Dassault Systèmes (2023). BIOVIA Discovery Studio Visualizer.

[62] Olsson M.H.M., Søndergaard C.R., Rostkowski M., Jensen J.H. (2011). PROPKA3: Consistent Treatment of Internal and Surface Residues in Empirical pKa Predictions. J. Chem. Theory Comput..

[63] Harris C.R., Millman K.J., van der Walt S.J., Gommers R., Virtanen P., Cournapeau D., Wieser E., Taylor J., Berg S., Smith N.J. (2020). Array programming with NumPy. Nature.

[64] Hunter J.D. (2007). Matplotlib: A 2D graphics environment. Comput. Sci. Eng..

[65] NCSS (2025). NCSS 2025 Statistical Software.

