# Supplementary material for: Selective Cytotoxic Activity of Derivatives of the Natural Alkaloid Tetrandrine Against Cancer Cell Lines: In Vitro and In Silico Studies
Source: Molecules. 2026 Oct 8;31(19):3572. doi: 10.3390/molecules31193572 (PMC13666725; doi:10.3390/molecules31193572)
Supplement: Supplementary file 1 [file molecules-31-03572-s001.zip › molecules-4488239-supplementary.pdf]

Supporting Information

Selective Cytotoxic Activity of Derivatives of the Natural Alkaloid Tetrandrine against Cancer Cell Lines: In Vitro and In Silico Studies

María Daniela Alvarez-Campa[1], Carmen María López-Saiz[1], Héctor Enrique Trujillo-Ruiz[1], Carmen Lizette Del-Toro-Sánchez[1], Juan Carlos Gálvez-Ruiz[2], Ramón Alfonso Moreno-Corral[3], Maribel Plascencia-Jatomea[1], Ariel Humberto Soto-Sánchez[3], Erika Silva-Campa[4], Martin S. Hernández-Zazueta[2,3]* and Karen Ochoa Lara[3]*

1 Departamento de Investigación y Posgrado en Alimentos, Universidad de Sonora, Rosales y Encinas s/n, Colonia Centro, Hermosillo 83000, Sonora, Mexico
2 Departamento de Ciencias Químico-Biológicas, Universidad de Sonora, Rosales y Encinas s/n, Colonia Centro, Hermosillo 83000, Sonora, Mexico
3 Departamento de Investigación en Polímeros y Materiales, Universidad de Sonora, Rosales y Encinas s/n, Colonia Centro, Hermosillo 83000, Sonora, Mexico;
4 Departamento de Física, Universidad de Sonora, Rosales y Encinas s/n, Colonia Centro, Hermosillo 83000, Sonora, Mexico

* Correspondence: martinsamuel.hernandez@gmail.com (M.S.H.-Z.); karen.ochoa@unison.mx (K.O.L.)

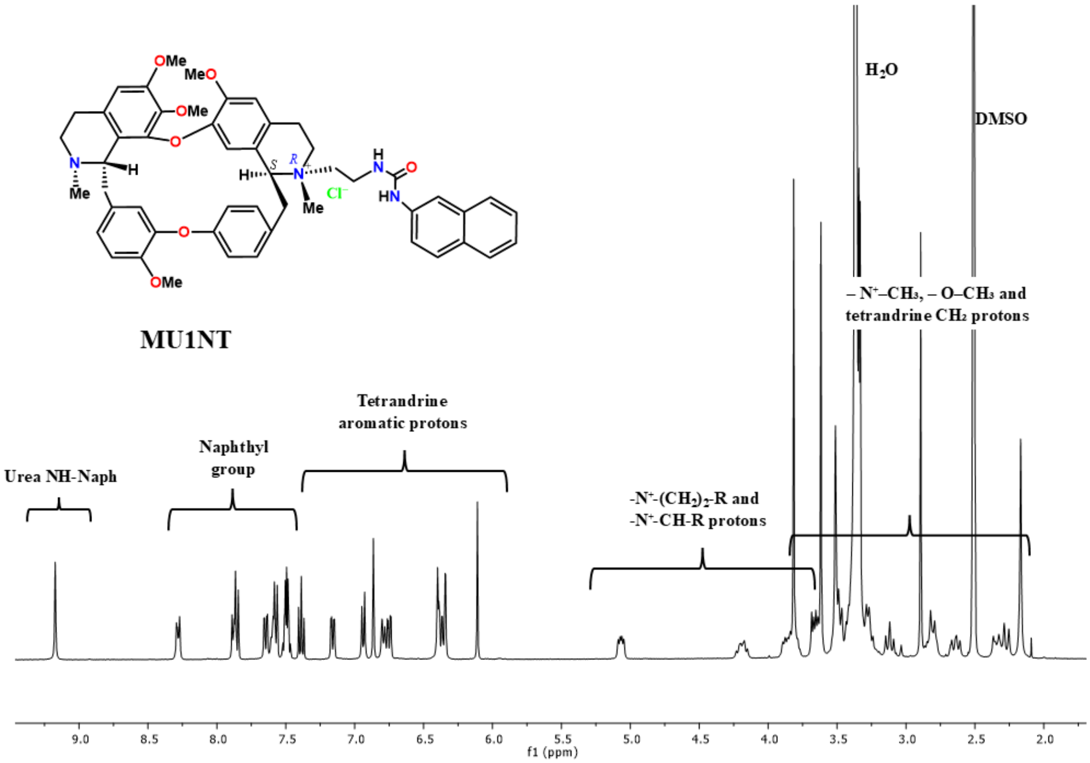

Figure S1. ^{1}H NMR spectrum of **MU1NT** in DMSO-d_6.

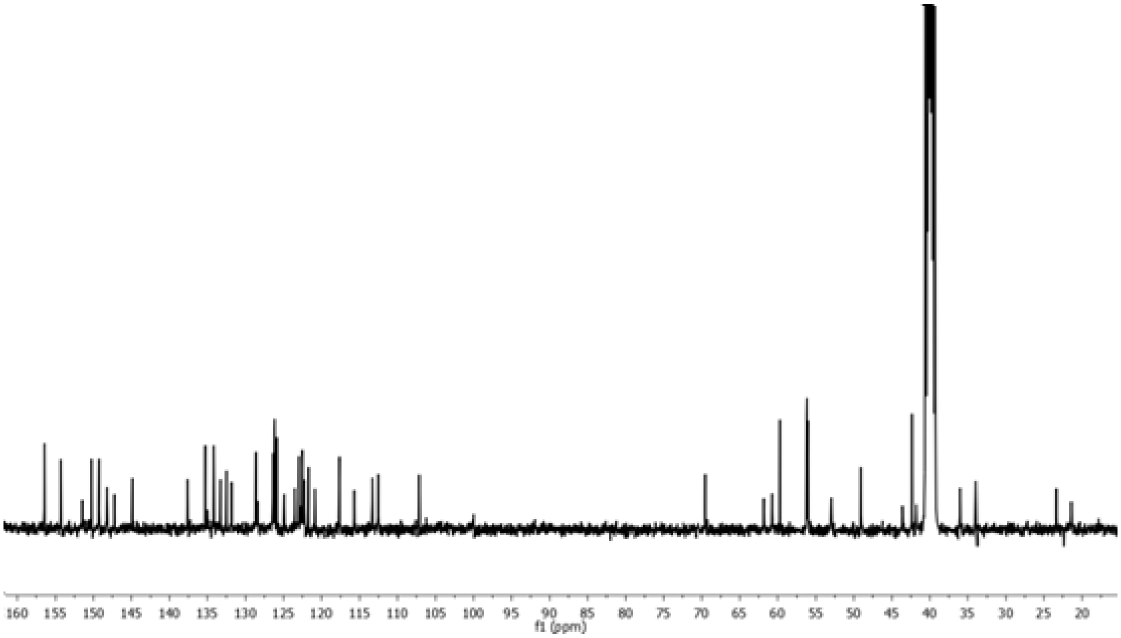

Figure S2. ^{13}C NMR spectrum of **MU1NT** in DMSO-d_6.

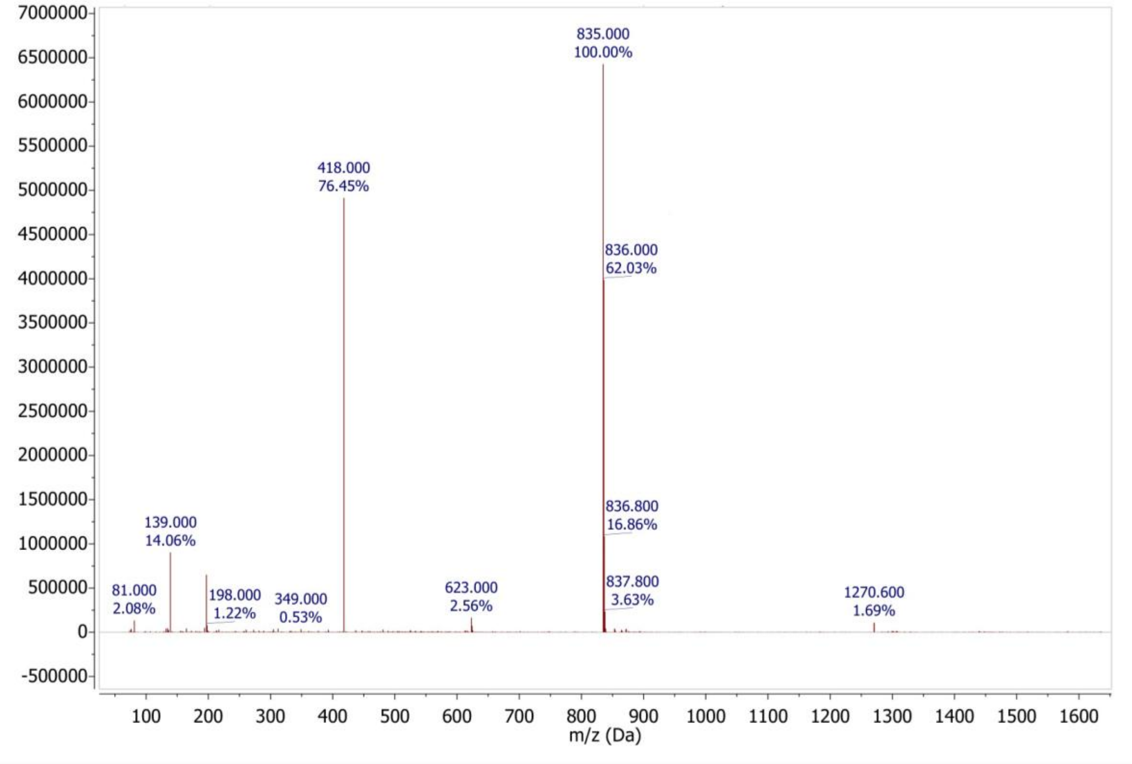

Figure S3. ESI(+) mass spectrum of **MU1NT**.

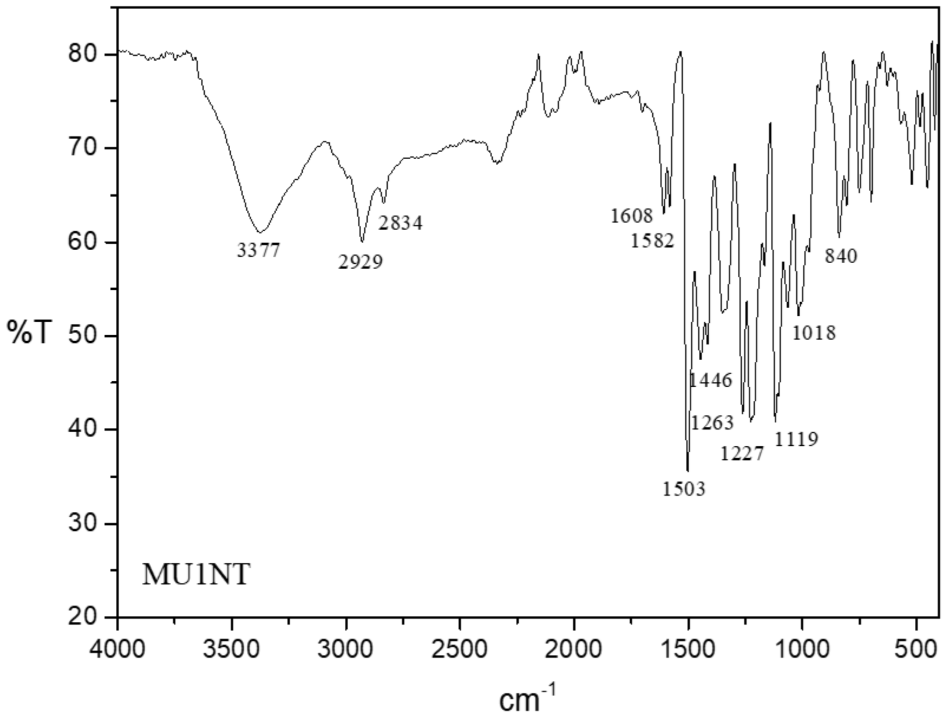

Figure S4. FTIR-ATR spectrum of **MU1NT**.

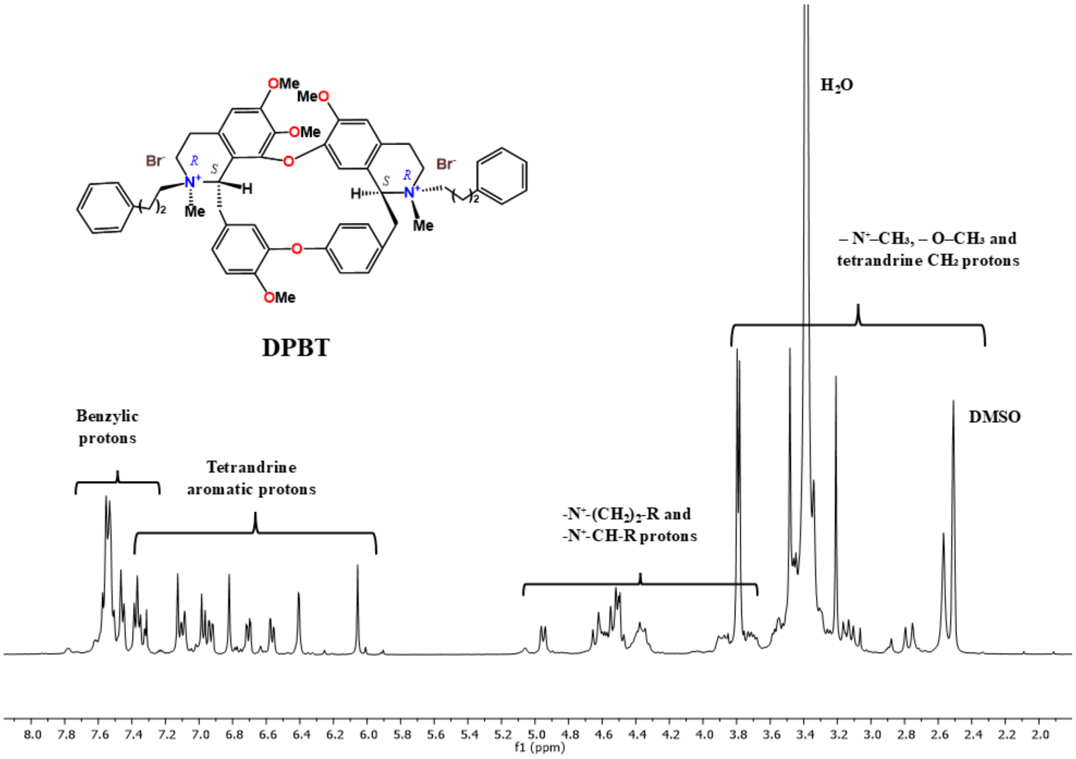

Figure S5. ^{1}H NMR spectrum of **DPBT** in DMSO-d_6.

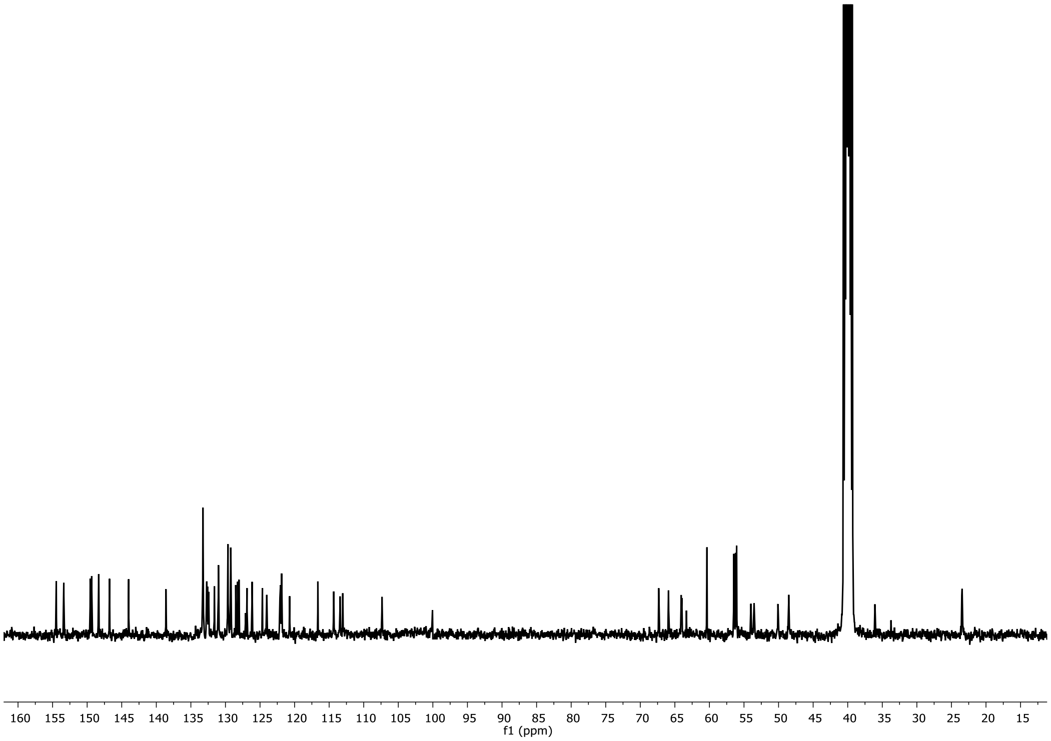

Figure S6. ^{13}C NMR spectrum of **DPBT** in DMSO-d_6.

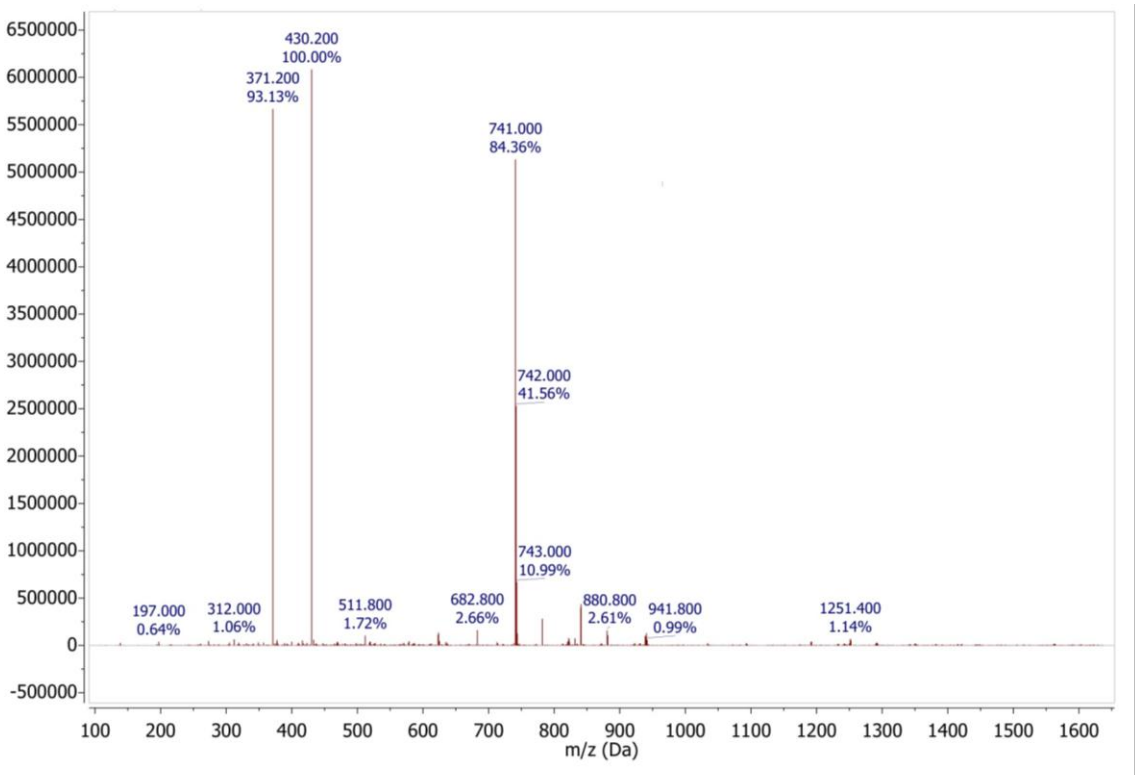

Figure S7. ESI(+) mass spectrum of **DPBT**.

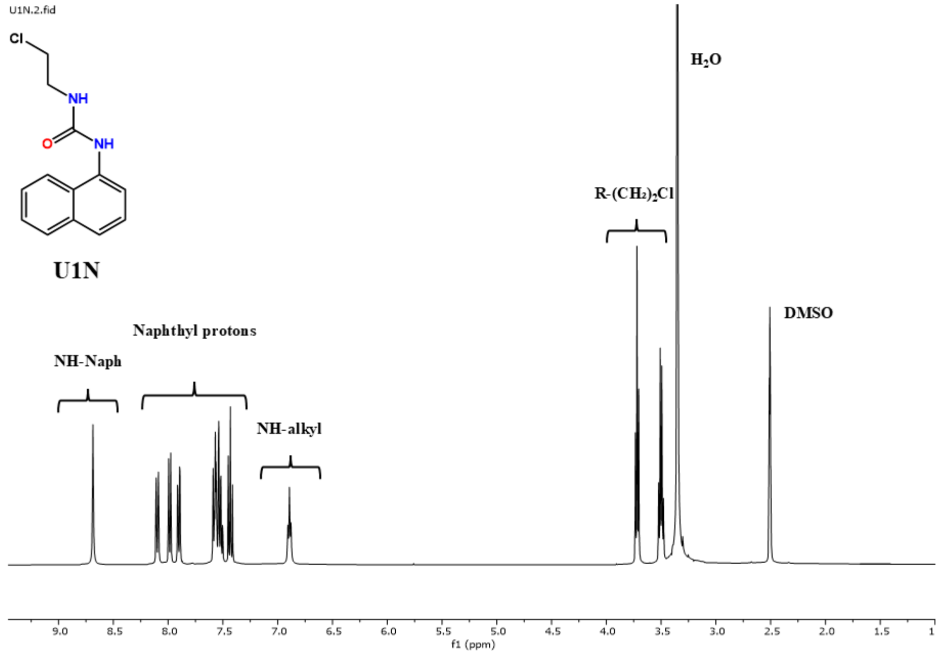

Figure S8. ^{1}H NMR spectrum of **U1N** in DMSO-d_6.

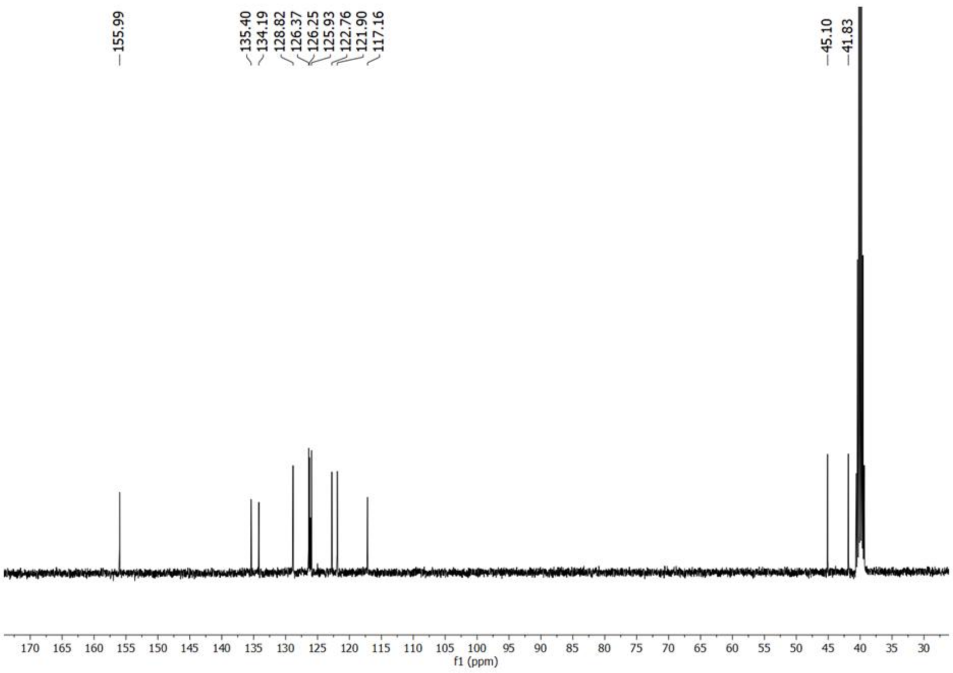

Figure S9. ^{13}C NMR spectrum of **U1N** in DMSO-d_6.

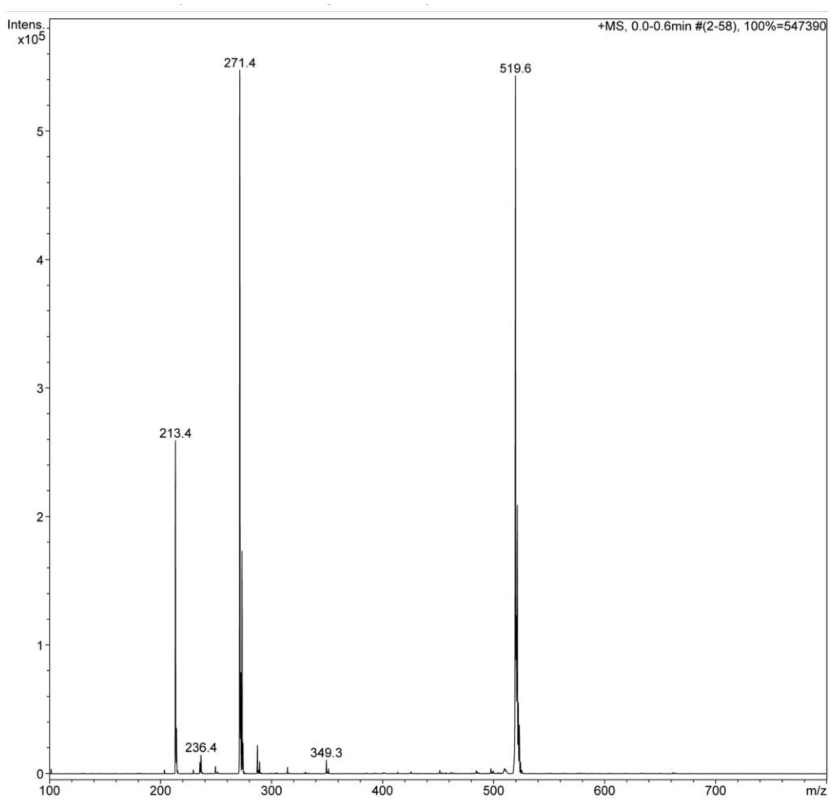

Figure S10. ESI(+) mass spectrum of **U1N**.

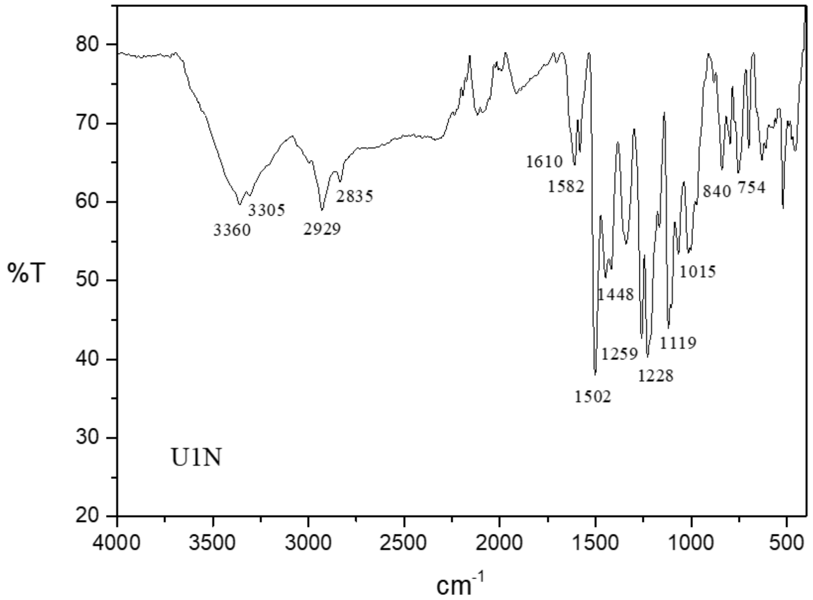

Figure S11. FTIR-ATR spectrum of **U1N**.

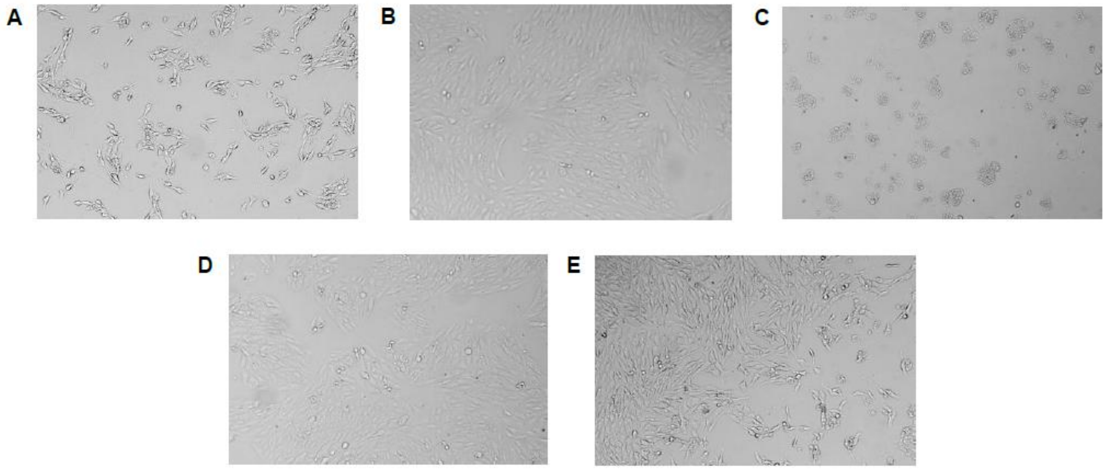

Figure S12. Representative bright-field microscopy images of ARPE-19 cells after 48 h of treatment with the selected compounds at 25 μM. **(A) TET**; **(B)** DMSO control; **(C)** doxorubicin; **(D) DBT**; **(E) MU1NT**.

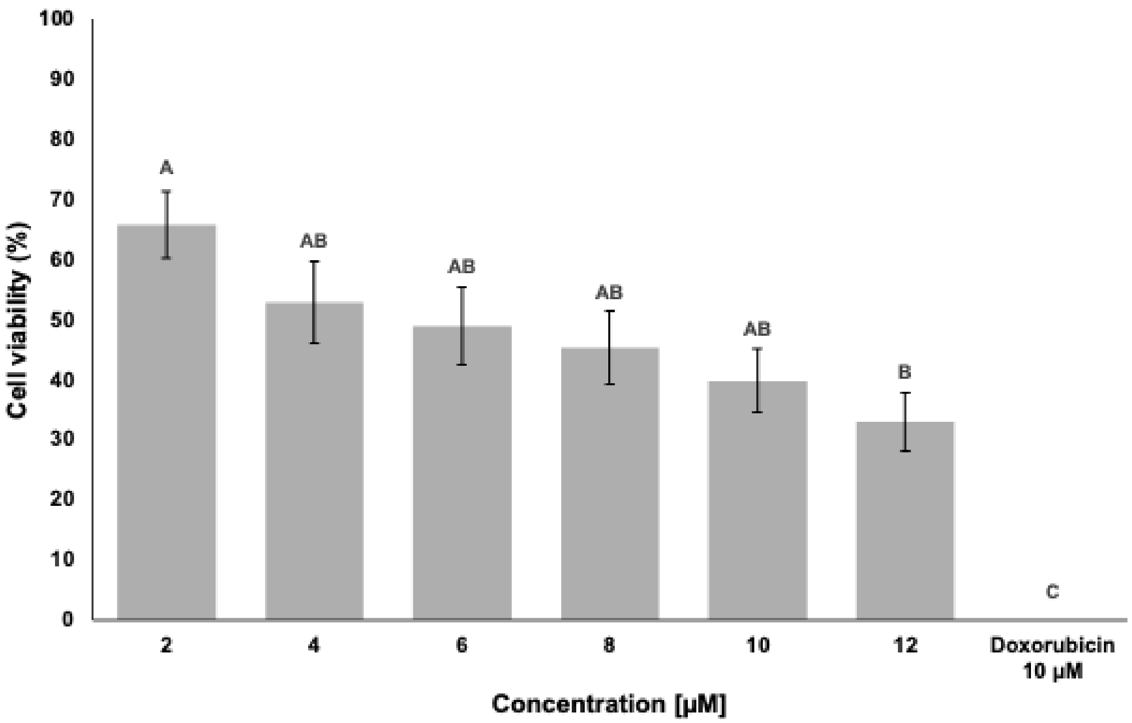

Figure S13. Representative concentration–response profile for **TET** in 22Rv1 cells after 48 h of exposure. Doxorubicin (10 μM) was included as a single-concentration positive cytotoxic control. Cell viability was determined using the MTT assay. Data are presented as the mean ± SEM of three independent experiments performed in triplicate. Bars sharing at least one letter are not significantly different, whereas bars with no letters in common are significantly different according to the Tukey–Kramer test ($p < 0.05$).

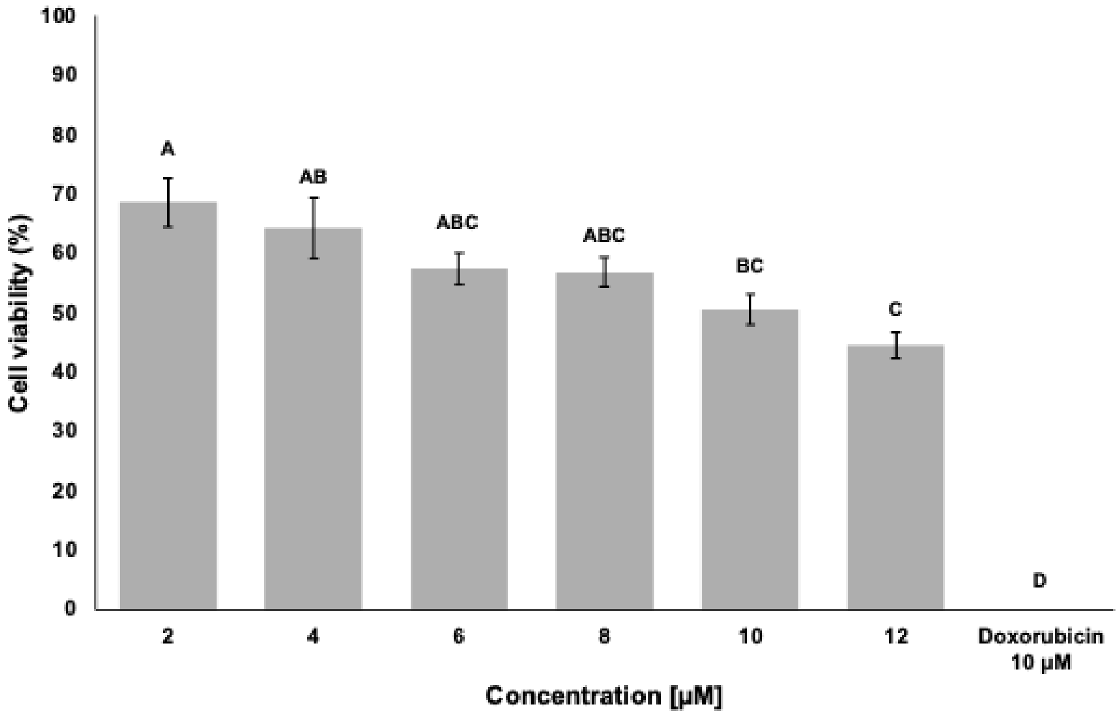

Figure S14. Representative concentration–response profile for **DBT** in 22Rv1 cells after 48 h of exposure. Doxorubicin (10 µM) was included as a single-concentration positive cytotoxic control. Cell viability was determined using the MTT assay. Data are presented as the mean ± SEM of three independent experiments performed in triplicate. Bars sharing at least one letter are not significantly different, whereas bars with no letters in common are significantly different according to the Tukey–Kramer test ($p < 0.05$).

Supplementary Method for Figure S15. Generation of the redocking-validation plot: Coordinate-frame overlays of the crystallographic and redocked venetoclax geometries were rendered directly from the output coordinates. Both poses were projected onto the principal axes of the crystallographic ligand, obtained by singular-value decomposition of its centered heavy-atom coordinate matrix, and plotted using Matplotlib 3.10.8 [64] and NumPy 2.4.6 [63]. The same coordinate transformation, derived from the crystallographic pose, was applied to both structures. No independent alignment or least-squares fitting was applied to the redocked pose.

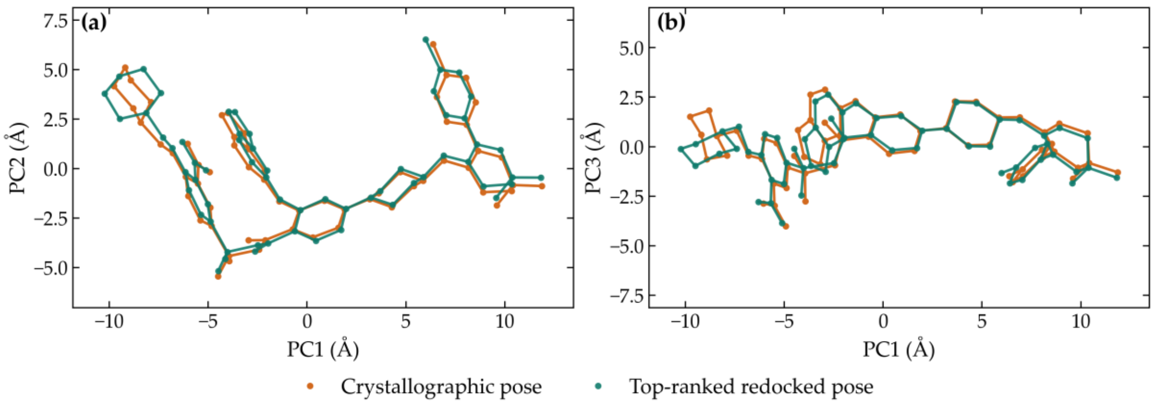

Figure S15. Validation of the targeted Bcl-2 docking protocol by redocking of venetoclax**.** The crystallographic conformation of venetoclax from PDB 6O0K (orange) is superimposed on the top-ranked redocked pose (green) and shown as two orthogonal projections onto the principal axes of the crystallographic ligand: (A) PC1–PC2 and (B) PC1–PC3. The symmetry-corrected heavy-atom RMSD was 0.55 Å, calculated in the receptor coordinate frame without post-docking fitting. Hydrogen atoms were omitted for clarity.

Table S1. Baseline RNA expression of apoptosis-related docking targets in the cancer cell lines used in the study (HPA v25.1; nTPM).

Gene/target	22Rv1	A549	MDA-MB-231	HCT-116	HeLa
**TNFRSF10A/DR4**	13.3	37.3	38.4	46.4	21.1
**TNFRSF10B/DR5**	29.7	109.8	85.4	62.3	41.8
**BCL2**	3.2	0.5	2.6	1.2	1.8
**BCL2L1/Bcl-XL**	20.3	165.9	360.6	197.7	42.5
**BAX**	59.0	240.9	187.7	90.9	67.1

Values are normalized transcripts per million (nTPM) from Human Protein Atlas version 25.1, Ensembl 109.

Table S2. Baseline RNA expression of docking targets in independent ARPE-19 controls from GEO GSE159235.

Gene/target	Median CPM	Range across four controls	Detected in all controls
TNFRSF10A/DR4	11.8	8.1-18.0	Yes
TNFRSF10B/DR5	167.5	137.6-180.3	Yes
BCL2	12.2	9.3-15.1	Yes
BCL2L1/Bcl-XL	100.0	34.1-210.8	Yes
BAX	39.0	18.5-127.8	Yes

CPM values from GEO GSE159235 are not numerically comparable with HPA nTPM values.
